# Visible‐Light‐Activated Multi‐Color Tunable Time‐Dependent Afterglow Triggered by Variable Conjugation Effects via the Transformation of Matrix

**DOI:** 10.1002/advs.202510317

**Published:** 2025-08-24

**Authors:** Hongpu Xin, Yunze Huang, Weibo Zhang, Peng Li, Huanrong Li

**Affiliations:** ^1^ Tianjin Key Laboratory of Chemical Process Safety Hebei Key Laboratory of Functional Polymers School of Chemical Engineering and Technology Hebei University of Technology Tianjin 300401 P. R. China

**Keywords:** carbon dots (CDs), multi‐color tunability, thermally activated delayed fluorescence (TADF), time‐dependent afterglow color (TDAC), visible light excitation, water‐stability

## Abstract

Achieving multi‐color tunable time‐dependent afterglow color (TDAC) in pure organic materials under visible light excitation remains a significant challenge. Herein, TDAC composites (CDs/U) are prepared with multi‐color tunability upon visible‐light excitation. Furthermore, the TDAC mechanism is the coexistence of shorter‐lived afterglow and longer‐lived afterglow. The long‐ and short‐wavelength afterglow in CDs/U originate from highly conjugated nitrogen heterocyclic structures and abundant surface functional states, respectively. More impressively, p‐CDs/U systems (p‐phenylenediamine (p‐PD) as carbon source) exhibit dynamic TDAC behaviors with different decay rates of long‐wavelength red afterglow as varying the reaction temperature varies. The experimental results indicate that the transformation of the matrix from biuret to ammelide and then to cyanuric acid (CA) decreases the conjugation degree of p‐CDs/U systems owing to the lower pyrrolic N content. This is not conducive to long‐wavelength emission, leading to shorter lifetimes in the long‐wavelength region. Meanwhile, the theoretical calculation confirms that the matrix is critical for efficient thermally activated delayed fluorescence (TADF). Moreover, the three composites exhibit distinct TDAC behaviors because p‐PD and o‐PD develop higher conjugation during CDs formation compared to m‐PD. Finally, benefiting from the excellent TDAC characteristics of these composites, we have successfully developed multi‐mode anti‐counterfeiting and multi‐dimensional encryption.

## Graphical abstract

Three carbon dots (CDs)‐based composites are synthesized via heat treatment of phenylenediamine derivatives with urea. These composites exhibit time‐dependent afterglow across most of the visible spectrum under visible‐light excitation. The matrix modulation strategy regulates the degree of conjugation in the composites, effectively tuning their afterglow properties. These features provide novel strategies for applications in dynamic anti‐counterfeiting and multi‐dimensional information encryption.

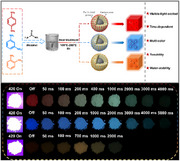

## 1 Introduction

Smart stimulus‐responsive room temperature afterglow (RTA) materials, whose optical properties can quickly respond to external stimuli such as light, heat, pH, and pressure,^[^ ^1^, ^2^, ^3^, ^4^ ^]^ have been extensively applied in the fields of information encryption, bio‐imaging, and optoelectronic devices.^[^ ^5^, ^6^, ^7^, ^8^, ^9^ ^]^ Especially, time‐dependent afterglow color (TDAC) can empower smart RTA materials with additional channels in the time dimension upon no external stimuli, which holds great potential for advanced applications in dynamic information encryption modes, multi‐mode anti‐counterfeiting, and high contrast molecular sensing. In recent years, several TDAC systems have been developed via designing the co‐existence of multiple emissive centers with diverse lifetimes using various strategies such as multi‐component doping,^[^ ^10^ ^]^ metal doping‐induced structure defects,^[^ ^11^ ^]^ aggregated phosphorescence etc.^[^ ^12^ ^]^ However, these materials frequently have tedious preparation processes, poor processability, and weak stability, which no longer meet the growing information storage demands. Furthermore, the majority of the above‐mentioned systems can only be activated by high‐energy UV light, which greatly restricts their extensive utilization owing to their substantial risk to human well‐being. In contrast, visible light possesses multiple advantages of high security to human health, deeper penetration capability, and practicability, which exhibit more practical application values. Therefore, it is very important to develop synthesis‐friendly visible light‐activated TDAC materials with good processability and high stability for expanding their application scope.

Carbon dots (CDs), as emerging quasi‐zero‐dimensional photoluminescent nanomaterials, have aroused significant interest for prospective applications owing to their multiple advantages, such as tunable optical properties, low toxicity, biocompatibility, high photostability, and easy synthesis.^[^ ^13^, ^14^, ^15^, ^16^, ^17^, ^18^ ^]^ In recent years, CDs‐based RTA materials with long lifetime, high efficiency and multicolor have been developed by two main strategies as follows: 1) introducing heteroatoms such as N, S, P, and B into CDs to facilitate the intersystem crossing (ISC) efficiency from the singlet states to the triplet states;^[^ ^19^, ^20^, ^21^, ^22^ ^]^ 2) embedding CDs into a rigid matrix, such as zeolite,^[^ ^23^ ^]^ silica,^[^ ^24^, ^25^, ^26^ ^]^ boric acid,^[^ ^27^, ^28^ ^]^ layered double hydroxides,^[^ ^29^, ^30^ ^]^ polyvinyl alcohol (PVA) etc.,^[^ ^31^, ^32^, ^33^ ^]^ to stabilize their triplet states through constraining the vibration and rotation of CDs by chemical bonds (such as covalent bonds and hydrogen bonds) and tight encapsulation. Unfortunately, most existing CD‐based RTA materials only present static afterglow output, which is unsuitable for advanced information technology applications. Therefore, it is necessary to exploit CD‐based TDAC materials for achieving time‐dimension information storage and promoting advanced dynamic anti‐counterfeiting. In 2021, Tan reported CDs‐based TDAC materials with dynamic tunable afterglow color from orange to green for the first time through the one‐pot hydrothermal treatment of levofloxacin.^[^ ^34^ ^]^ Furthermore, several efficient TDAC systems with full color, longer lifetimes, and higher efficiency have been developed via embedding diverse CDs (e.g., o‐phenylenediamine derivatives and surface‐rich carboxyl groups, etc.) into various matrices (e.g., urea, PVA, boric acid (B_2_O_3_), and hydrothermal B_2_O_3_ (H‐B_2_O_3_)), crosslink enhanced emission (CEE) effects, and modified solid‐phase pyrolysis routes. Nevertheless, the reports on CD‐based TDAC materials are still relatively few so far due to the lack of in‐depth understanding about the production mechanisms of TDAC property and controllable wavelength changes. More importantly, the vast majority of them can only be limited to UV light activation. As is well known, visible light has higher security, deeper penetration capability, and better practicability. Therefore, new strategies are necessary to develop novel visible light‐activated TDAC materials for more broader application prospects.

Herein, via a simple heat treatment of phenylenediamine (PD) derivatives and urea, we successfully prepared TDAC materials with diverse properties upon visible‐light excitation. Their afterglow colors almost cover the entire visible range, exhibiting color transitions from red to green, blue to cyan, and red to orange. Impressively, with varying the reaction temperature, p‐CDs/U systems (p‐CDs/U@X) exhibit dynamic TDAC behaviors with different decay rates of red afterglow, achieving fine‐tuning of the afterglow color. Furthermore, we elucidate the underlying mechanisms of TDAC; the long‐wavelength emissions originate from the highly conjugated nitrogen heterocyclic structure, and the short‐wavelength emissions stem from the abundant surface functional groups. By controlling the reaction temperature, which governs the transformation of the matrix from biuret to ammelide and then to cyanuric acid (CA), the TDAC properties can be effectively regulated. Additionally, the distinct TDAC behaviors of the three composites are attributed to the different mechanisms of CDs formation. Meanwhile, theoretical calculations based on density functional theory (DFT) demonstrate the critical role of the matrix in achieving efficient thermally activated delayed fluorescence (TADF). Based on the unique properties of CDs/U composites, we developed multi‐mode anti‐counterfeiting and multi‐dimensional information encryption technologies. To date, CDs‐based materials with the TDAC phenomenon under visible light excitation are rarely reported, which provides novel ideas and directions for the practical application in advanced dynamic anti‐counterfeiting and multi‐dimensional information encryption.

## 2 Results and Discussion

**Figure 1:**
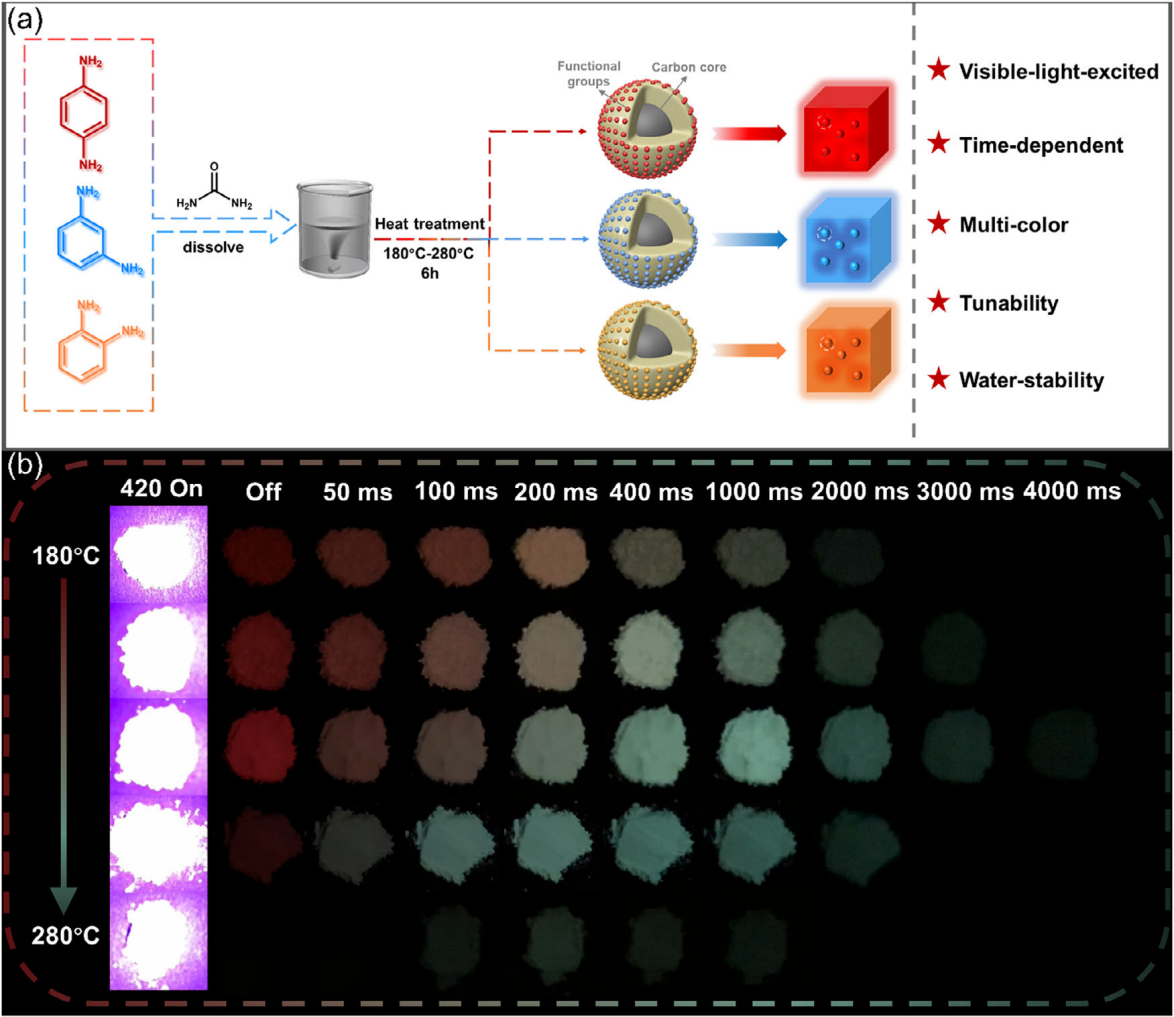
a) Schematic diagram of the synthesis process of CDs/U composites. b) Digital photographs of p‐CDs/U@X composites under 420 nm light irradiation and after being turned off.

A series of CD‐based afterglow materials was prepared by heat treatment at different temperatures using p‐phenylenediamine (p‐PD), m‐phenylenediamine (m‐PD), and o‐phenylenediamine (o‐PD) as precursors and urea as the matrix (**Figure** **1**a), which were named as p‐CDs/U@X, m‐CDs/U@X, and o‐CDs/U@X, respectively (where X represents the temperature). Unexpectedly, when excited by visible (Vis) light with a wavelength of 420 nm, all three systems present dynamic color‐tunable time‐dependent afterglow properties. Moreover, the time‐dependent afterglow colors are closely related to the reaction temperature. As illustrated in Figure 1b, p‐CDs/U@180 exhibits a gradual afterglow color change from red [Vis off (t_off_) = 0 ms] to green (t_off_ = 400 ms). As the reaction temperature increases from 180 to 280 °C, the red afterglow fades away more rapidly, substituted by green afterglow (Movie S1, Supporting Information). Similarly, m‐CDs/U@X and o‐CDs/U@X composites display color variation from blue to cyan, and red to orange with different rates respectively as the increased reaction temperature from 180 to 280 °C (Figure S1, Supporting Information). Thereby, the aforesaid three systems exhibit dynamic afterglow color transitions from red to green, blue to cyan, and red to orange, covering most of the entire visible region (Movie S2, Supporting Information). Moreover, these afterglow features show excellent water stability (Figures S2–S4, Movie S3, Supporting Information).

**Figure 2:**
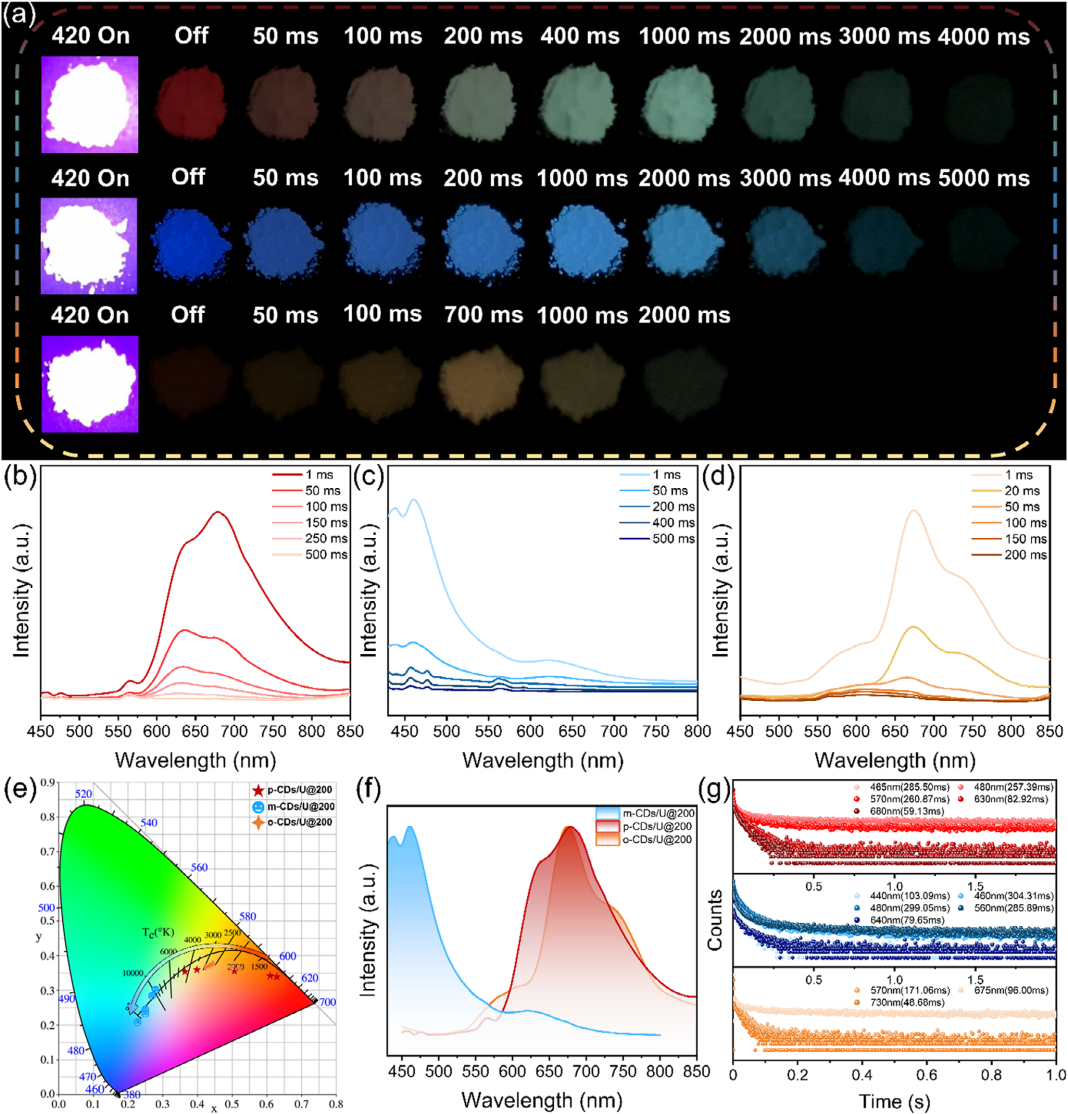
a) Digital photographs of p‐CDs/U@200, m‐CDs/U@200, and o‐CDs/U@200 under 420 nm light irradiation and after being turned off, respectively. TRES spectra of (b) p‐CDs/U@200, c) m‐CDs/U@200, and d) o‐CDs/U@200. e) CIE coordinate diagrams corresponding to the TRES spectra of the three composites. f) Normalized afterglow emission spectra of three composites. g) Afterglow decay curves of p‐CDs/U@200, m‐CDs/U@200, and o‐CDs/U@200 composites monitored at their respective emission centers.

To investigate the time‐dependent afterglow feature in the above‐mentioned composites, we used CDs/U@200 (**Figure** **2**a) as representatives and measured their time‐resolved emission spectra (TRES) under excitation at 420 nm (Figure 2b–d). For p‐CDs/U@200, with a delay time of 1 ms, the afterglow emission spectrum displays five emission peaks at 465 nm, 480 nm, 570 nm, 630 nm, and 680 nm with the maximum peak at 680 nm, exhibiting the short‐lived red afterglow with the Commission Internationale de l’ Eclairage (CIE) coordinates of (0.63, 0.34). As time elapses, the maximum emission peak gradually shifts from 680 nm to 630 nm, accompanied by the replacement of red color by a long‐lived green afterglow (Figure 2b). The corresponding CIE coordinates also shift from (0.63, 0.34) to (0.37, 0.35) (Figure 2e). As for m‐CDs/U@200, the TRES spectrum shows two dominant emission peaks at 440 and 460 nm with a delay time of 50 ms. Over time, the peak at 440 nm completely disappeared concomitant with the emergence of a new peak at 480 nm, resulting in a color change from blue to cyan (Figure 2c). While its CIE coordinates gradually change from (0.23, 0.21) to (0.28, 0.30) (Figure 2e). In o‐CDs/U@200, the broad spectrum with a main band at 675 nm and a shoulder peak at 730 nm gradually shifts toward a broad band at 570 nm, bringing about a color change from red to orange (Figure 2d). Meanwhile, the CIE coordinates show a transition from (0.52, 0.37) to (0.43, 0.37) (Figure 2e). The normalized afterglow emission spectra of CDs/U@200 are shown in Figure 2f; the different emission centers almost cover the visible spectrum. Evidently, CDs/U@200 present the ability of tunable afterglow colors covering most of the visible range under visible light excitation of 420 nm, which has not been reported so far.

Furthermore, the lifetimes of CDs/U@200 were measured to in‐depth understand their time‐dependent afterglow feature (Figure 2g). For p‐CDs/U@200, the average lifetimes were 285.50, 257.39, 260.87, 82.92, and 59.13 ms monitored at 465, 480, 570, 630, and 680 nm, respectively, implying that the coexistence of shorter‐lived red afterglow and longer‐lived blue and yellow afterglow results in a dynamic color change from red to green. In the case of m‐CDs/U@200, the average lifetimes were found to be 103.09 ms at 440 nm, 304.31 ms at 460 nm, 299.05 ms at 480 nm, 285.89 ms at 560 nm, and 79.65 ms at 640 nm. This indicates that the blue color decays faster than the cyan color, leading to a transition of afterglow color from blue to cyan. As for o‐CDs/U@200, the average lifetimes of afterglow emission at 570, 675, and 730 nm were measured as 171.06, 96.00, and 48.68 ms, respectively. This illustrates that the orange color persists longer compared with red, thus resulting in a color transition from red to orange.

Overall, the dynamic afterglow colors of CDs/U with time‐dependent feature under visible‐light excitation of 420 nm originate from the proportion variations of emission centers, which are related to the delay time. Differently, none of the three composite materials exhibited time‐dependent afterglow when excited by UV light of 254, 302, and 365 nm (Figure S5, Supporting Information). This phenomenon may be because the main emission peak in their afterglow emission spectra exhibits significantly higher intensity and longer lifetime compared to other peaks when the material is excited by these wavelengths, resulting in a single static afterglow (Figure S6, Supporting Information).

To further elucidate the TDAC mechanism, temperature‐dependent afterglow emission spectra of p‐CDs/U@200, o‐CDs/U@200 and m‐CDs/U@200 were conducted. As shown in Figures S7–S9 (Supporting Information), the afterglow intensity of all delayed emission peaks presents an increase with temperature, implying that these emission centers are ascribed to TADF rather than RTP. The photoluminescence quantum yields (PLQYs) of p‐CDs/U@200, m‐CDs/U@200, and o‐CDs/U@200 under 420 nm excitation are measured as 18.02%, 22.25%, and 39.40%, respectively. Furthermore, the afterglow quantum yields (AQYs) of 8.69%, 11.01%, and 31.11%, respectively (Table S1, Supporting Information). These values are higher than those of many CDs under visible light excitation (Table S2, Supporting Information). Additionally, the impact of different phenylenediamine and urea ratios on the afterglow properties of CDs/U composites was investigated. As shown in Figures S10 and S11 (Supporting Information), the intensity and lifetimes of the afterglows reach their maximum values when the mass ratio of p‐PD and urea is 1:150. This suggests that an excessively high precursor concentration is not favorable for afterglow emission.

**Figure 3:**
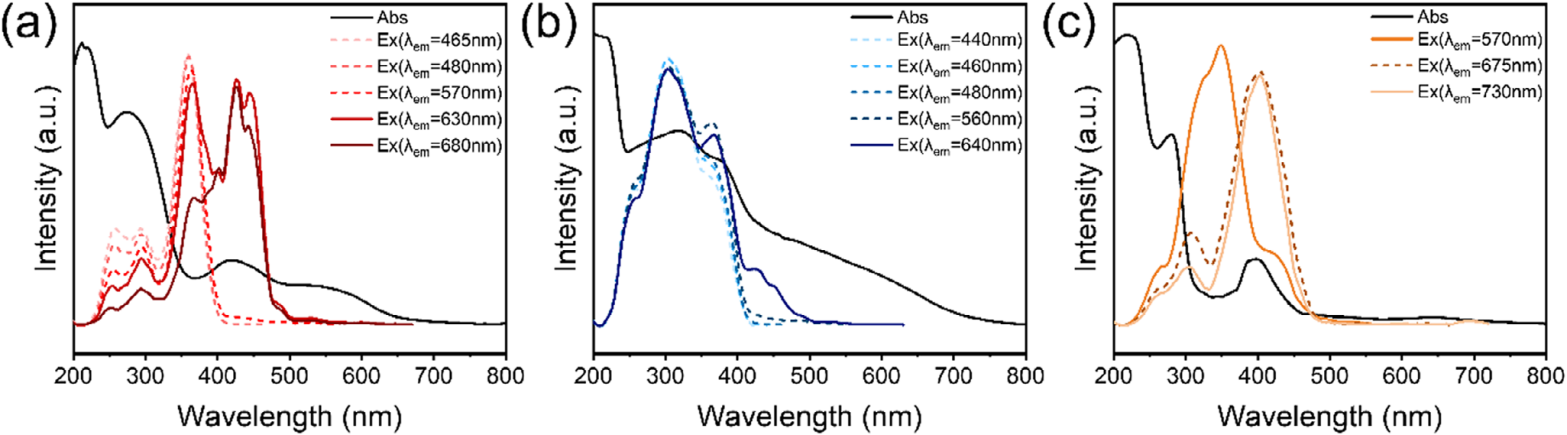
UV–vis absorption spectrum and afterglow excitation (monitored at different emission wavelengths) spectra of a) p‐CDs/U@200, b) m‐CDs/U@200, and c) o‐CDs/U@200.

To identify the afterglow source, we investigated the photophysical properties of three composites (**Figure** **3**a–c). For p‐CDs/U@200, the UV–vis absorption spectrum displays an absorption band at 215 nm attributed to the *π*–*π* ^*^ transition of the aromatic sp^2^ domains. And the wide absorption band from 250 to 500 nm indicates the generation of abundant excited states containing n‐π^*^ transition of C═O/C═N in surface state (250–370 nm) and the conjugated nitrogen heterocyclic structure of p‐CDs (370 nm‐500 nm). The afterglow emission of p‐CDs/U@200 at 680 and 630 nm can be excited by the absorption band centered at 425 nm, which is in good accordance with the absorption band within the range of 370–500 nm, indicating that the red afterglow originates from the highly conjugated structure of p‐CDs.Yet their excitation spectrum displays a distinct peak at 365 nm when monitoring at 570, 480, and 465 nm, which overlap well with their absorption band from 250 to 370 nm, suggesting that the green afterglow is closely related to the abundant surface states (Figure 3a). The conjugated nitrogen heterocyclic structure refers to the carbon cores‐based conjugated structure of p‐CDs, and the surface states refer to the surface‐rich groups on the conjugated nitrogen heterocyclic structure of p‐CDs (Scheme S1, Supporting Information).^[^ ^34^, ^35^ ^]^ For o‐CDs/U@200, the optimal excitation wavelength is 340 nm at a monitored wavelength of 570 nm, overlapping with the weak absorption band associated with surface states. When the emission wavelengths changed to 675 and 730 nm, the predominant excitation peaks are both located at 395 nm, coinciding with the absorption band at 400 nm, which is also related to the conjugated nitrogen heterocyclic structure of CDs (Figure 3c). Differently, for m‐CDs/U@200, the optimal excitation at different emission wavelengths is consistently 310 nm, coinciding with the absorption band at 315 nm, which indicates that these luminescent centers are attributed to the n‐π^*^ transition of the surface states (Figure 3b). Thus, the conjugated nitrogen heterocyclic structure, formed by crosslinking between the matrix and CDs, is responsible for the long‐wavelength emission. While the surface states of CDs are dominated by C═O/C═N groups, which are responsible for the shorter‐wavelength emissions.

**Figure 4:**
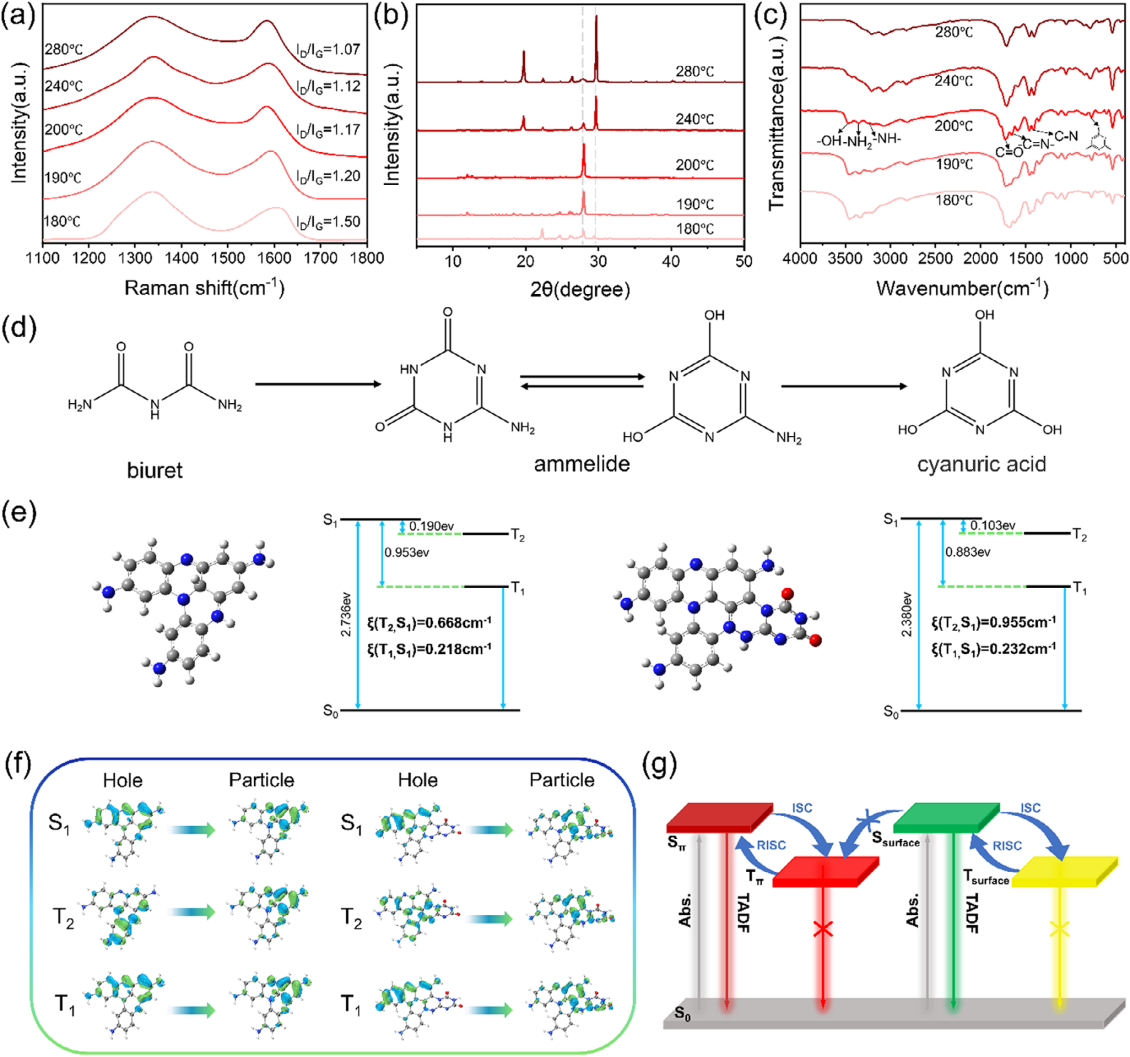
a) Raman spectra of p‐CDs/U@X composites under 514 nm excitation. b) XRD patterns of p‐CDs/U@X composites. c) FT–IR spectra of p‐CDs/U@X composites. d) Schematic diagram of matrix transformation process. e) Energy‐level difference diagrams and SOC constants calculated by p‐CDs and p‐CDs/U@200 system. f) Natural transition orbitals (NTOs) for S_1_, T_2_, and T_1_ states of p‐CDs and p‐CDs/U@200 system. g) Possible TADF emission mechanisms of p‐CDs/U@200 composites.

To gain more insight into the relationship between TDAC properties and structural components of CDs/U, we take p‐CDs/U@X for example, to measure their afterglow features, morphologies, and structures. Transmission electron microscopy (TEM) images show that p‐CDs/U@180 exhibit spherical particles with an average size of 1.28 nm, uniformly distributed in the matrix. Along with the temperature going up, the average size of the spherical particles gradually increases from 1.28 to 7.25 nm (Figures S12 and S13, Supporting Information). High‐resolution TEM (HR‐TEM) shows no obvious lattice fringes in p‐CDs/U@180 samples, implying their amorphous structures. While clear lattice fringes with an interlayer spacing of 0.25 nm for the (1120) graphene planes can be observed in p‐CDs/U@X (X = 190–280 °C) (Figure S14, Supporting Information), indicating their crystalline structures.^[^ ^22^ ^]^ Additionally, Raman spectra of p‐CDs/U@X exhibit two typical characteristic peaks at 1335 and 1583 cm^−1^, which are attributed to the disordered sp^3^ carbon (D band) and graphitic carbon network (G band), respectively. We found that the relative intensity ratios (I_D_/I_G_) gradually decrease with increasing reaction temperature, demonstrating an enhancement in graphitization degree (**Figure** **4**a).^[^ ^27^ ^]^ Normally, the increased size and carbonization degree of CDs are conducive to the afterglow emission redshift or the production of the longer‐wavelength emissions because the enlargement in the conjugated regions can effectively narrow the energy‐level difference between the excited singlet state (S_1_) and the excited triplet state (T_1_).^[^ ^5^ ^]^ However, this is contrary to the variation rule of afterglow emission in p‐CDs/U@X. As shown in Figure S15 (Supporting Information), although p‐CDs/U@X show identical emission centers, the relative intensity of the longer‐wavelength emissions and the shorter‐wavelength emissions gradually declines with increasing reaction temperature. Furthermore, their relative lifetimes also display similar changes (Figure S16, Supporting Information). In view of this, we speculate that the urea matrix may play a crucial role in their unique afterglow feature.

To verify this inference, X‐ray diffraction (XRD) patterns and Fourier transform‐infrared (FT‐IR) spectra of p‐CDs/U@X were measured (Figure 4b,c). As illustrated in Figure 4b, p‐CDs/U@180 presents four typical peaks of biuret appearing at 18.4°, 21.0°, 24.7°, and 26.1°. As reaction temperature increases to 190 °C, the characteristic peaks belonging to biuret gradually disappear, while the characteristic peaks at 28° assigned to ammelide rapidly increase.^[^ ^36^ ^]^ For p‐CDs/U@200, XRD patterns almost completely show the characteristic peaks of ammelide (Figure S17a, Supporting Information). With the continuous increase of the reaction temperature, the characteristic peaks of ammelide in p‐CDs/U@240 and p‐CDs/U@280 significantly weaken, while the peaks at 17.3°, 19.5°, 19.8°, 22.5°, 26.3°, 28.3°, and 29.8° attributed to CA rapidly intensify (Figure S17b, Supporting Information). These XRD patterns demonstrate the urea pyrolysis transformation pathway involving urea‐biuret‐ammelide‐CA, which can also be supported by the FT‐IR and Raman spectra of p‐CDs/U@X.

In the FT‐IR spectra of p‐CDs/U@180, p‐CDs/U@190, and p‐CDs/U@200, the peaks at 3467, 3352, 3250, 1730, 1654, and 775 cm^−1^ correspond to the stretching vibrations of ─OH, ─NH_2_, ─NH─, C═O, ─C═N─, and the triazine ring, respectively. And the peaks at 1461 and 1411 cm^−1^ were attributed to the stretching vibrations of C─N (Figure 4c).^[^ ^36^, ^37^, ^38^, ^39^ ^]^ These peaks almost overlapped with those of ammelide (Figure S18a, Supporting Information). The slight peak differences around 1510–1780 cm^−1^ occur because part of the biuret in p‐CDs/U@180 and p‐CDs/U@190 does not convert to ammelide. For p‐CDs/U@240 and p‐CDs/U@280, the characteristic peaks at 3352 cm^−1^ (corresponding to ─NH_2_) gradually disappear. While the intensity of peaks at 3213 cm^−1^ (corresponding to ─NH─) and 1710 cm^−1^ (corresponding to C═O) increases significantly. The FT–IR spectra show complete overlap with those of CA, with only minor differences attributable to the presence of CDs. This indicates complete conversion of the matrix to CA (Figure S18b, Supporting Information). Furthermore, Raman spectra of p‐CDs/U@X verify the above viewpoint. As shown in Figure S19 (Supporting Information), p‐CDs/U@X exhibits a characteristic peak at 700 cm^−1^ corresponding to the triazine ring. The weaker peak intensity of p‐CDs/U@180 was attributed to only partial conversion of biuret to ammelide. With increasing reaction temperature, the triazine ring content increased, reflected by the rising peak intensities. For p‐CDs/U@280, however, the triazine ring peak intensity decreased, which may be attributed to thermal decomposition of CA, as evidenced by the thermogravimetric analysis (TGA) curve of CA (Figure S20, Supporting Information). The observed mass loss of CA at 230–255 °C may be attributed to the release of both adsorbed water and water of crystallization through evaporation.^[^ ^40^ ^]^ While in p‐CDs/U@280, the water is completely evaporated by the heating process. Furthermore, owing to the abundant hydrogen bonds between CDs and the CA matrix, the composite p‐CDs/U@280 exhibits higher thermal stability than pure CA. Thus, p‐CDs/U@280 exhibited no significant mass loss before 300 °C. These results demonstrate that with increasing reaction temperature, the matrix transforms from biuret to ammelide and ultimately to CA (Figure 4d).

To understand in greater depth the influence of urea‐evolved various matrices on the afterglow properties of p‐CDs/U@X, X‐ray photoelectron spectroscopy (XPS) of p‐CDs/U@X was measured. Their high‐resolution XPS N 1s spectra reveal that the pyrrolic N content gradually decreases from 38.22% to 21.01% as the reaction temperature increases (Figure S21, Table S3, Supporting Information), indicating the generation of nitrogen heterocyclic structures with decreased degrees of conjugation in matrix transformations from biuret to ammelide and to CA. This result is because biuret and ammelide possess a higher number of amino groups compared with CA, which can better crosslink with the ‐NH‐ on the CDs surface, leading to the formation of more pyrrolic N. Based on this, p‐CDs/U@X have the higher content of the longer‐wavelength emissions with the lower reaction temperature, which are responsible for their diverse TDAC properties.

Theoretical calculations based on density functional theory (DFT) were performed to analyze the critical role of the matrix in achieving efficient TADF emission for p‐CDs/U using p‐CDs/U@200 as an example (Figure 4e). Previous studies have investigated the formation mechanisms of CDs from amine derivatives.^[^ ^41^, ^42^ ^]^ Based on these existing studies and the presence of ammelide in p‐CDs/U@200, we performed modeling of p‐CDs and p‐CDs/U@200 (Discussion S1 and Figure S22, Supporting Information). According to the aforementioned experimental results, the afterglow type is ascribed to TADF. However, the energy‐level difference (ΔE_S1T1_) between the lowest singlet excited state (S_1_) and the lowest triplet excited state (T_1_) of p‐CDs/U@200 is as high as 0.883 eV, making reverse intersystem crossing (RISC) from T_1_ to S_1_ nearly impossible in theory. Subsequently, we found that the energy‐level difference (ΔE_S1T2_) between the triplet states (T_2_) and the singlet states (S_1_) of p‐CDs/U@200 is 0.103 eV, which is the best value for the TADF phenomenon.^[^ ^22^ ^]^ This clearly indicates that the TADF phenomenon of p‐CDs/U@200 originates from the sufficiently small ΔE_S1T2_ between T_2_ and S_1_. Furthermore, we compared the corresponding data of p‐CDs and p‐CDs/U@200. In p‐CDs/U@200 system, the matrix and the surface functional group of p‐CDs crosslink through N─N bonds. For p‐CDs/U@200, compared to p‐CDs, the energy‐level difference between T_2_ and S_1_ decreases from 0.190 to 0.103 eV, while the spin‐orbit coupling (SOC) constant ξ (T_2_, S_1_) increases from 0.668 to 0.955 cm^−1^. This clearly shows that the RISC process is becoming easier for p‐CDs/U@200 due to the narrower ΔE_ST_ and increased SOC constant. Figure 4f displays the natural transition orbitals (NTOs) of the p‐CDs and p‐CDs/U@200 systems, which illustrate the distributions of holes and particles in the S_1_, T_2_, and T_1_ states. We found that only in the p‐CDs/U@200 system did the holes and particles, both in the T_2_ state, partially overlap. For the T_2_ state, the hole almost spreads over the whole molecule skeleton, while the particle is distributed at the junction between the matrix and CDs. The partial overlap between the hole and the particle reveals hybrid local and charge transfer (HLCT) character in the T_2_ state,^[^ ^43^, ^44^, ^45^ ^]^ which may play an important role in the RISC process.^[^ ^46^, ^47^ ^]^

Based on the above experimental data and theoretical calculations, a possible mechanism for afterglow emission of p‐CDs/U@X is proposed (Figure 4g). Upon irradiation with 420 nm visible light, conjugated structures and surface states are concurrently activated. The conjugated nitrogen heterocyclic structures are responsible for longer‐wavelength emissions, while the surface states are accountable for shorter‐wavelength emissions. In the nitrogen heterocyclic structure, the ground state (S_0_) electrons undergo excitation to the singlet excited‐state (S_n_), which rapidly relaxes to the lowest singlet excited‐state (S_π_). Part of the S_π_ state electrons will reach the lowest triplet excited‐states (T_π_) via intersystem crossing (ISC) and internal conversion (IC), then transition back to the S_π_ state through RISC process, and ultimately return to the S_0_ from the S_π_ state to produce a fast‐decaying red afterglow. For surface states, the lowest singlet excited‐state (S_surface_) electrons also reach the lowest triplet excited‐states (T_surface_) via the ISC and IC, then return to the S_surface_ state through RISC, from which they then transition back to the S_0_ state, leading to slow‐decaying green afterglow.

To interpret the water stability of the afterglow feature for p‐CDs/U@X, we prepared their water solution and conducted in‐depth research. As illustrated in Figure S23 (Supporting Information), the FT‐IR spectra of p‐CDs/U@200 and p‐CDs/U@200‐Water reveal that after the addition of water, the characteristic O─H absorption peak is gradually broader and shifts from 3467 to 3425 cm^−1^, and the characteristic C═O absorption peak shifts from 1730 to 1698 cm^−1^ (Figure S23a, Supporting Information). These changes indicate stronger hydrogen bonding interactions in the presence of water.^[^ ^37^, ^48^, ^49^ ^]^ Raman spectroscopy further supported the conclusions derived from FT‐IR spectroscopy. Upon water addition, the characteristic peaks at 700 and 1673 cm^−1^ showed significant shifts, suggesting strong hydrogen bonding (Figure S23b, Supporting Information).^[^ ^50^ ^]^ Furthermore, differential scanning calorimetry (DSC) of a series of p‐CDs/U@200 with different water contents was measured (Figure S24, Supporting Information), which indicates that the above hydrogen bonding may be a hydrogen‐bonded network. At a low water content (20%), only a faint endothermic melting peak was detected. As the water content increased from 20% to 40%, the endothermic peak intensified and shifted significantly toward higher temperatures, suggesting the presence of two states of freezing water (freezing bound water and bulk water) and saturated non‐freezing bound water in the composite.^[^ ^51^ ^]^ Meanwhile, the exothermic signals became stronger with increasing water content, attributed to the rise in bulk water. Thus, in samples with less than 20% water content, all water likely exists as non‐freezing bound water. The non‐freezing bound water can construct robust hydrogen‐bonded networks, which more effectively prevent water and dissolved oxygen from quenching triplet excitons in liquid environments.^[^ ^49^ ^]^ These results demonstrate that the formation of hydrogen bond networks in p‐CDs/U@200 when immersed in water endows it with excellent water stability.

**Figure 5:**
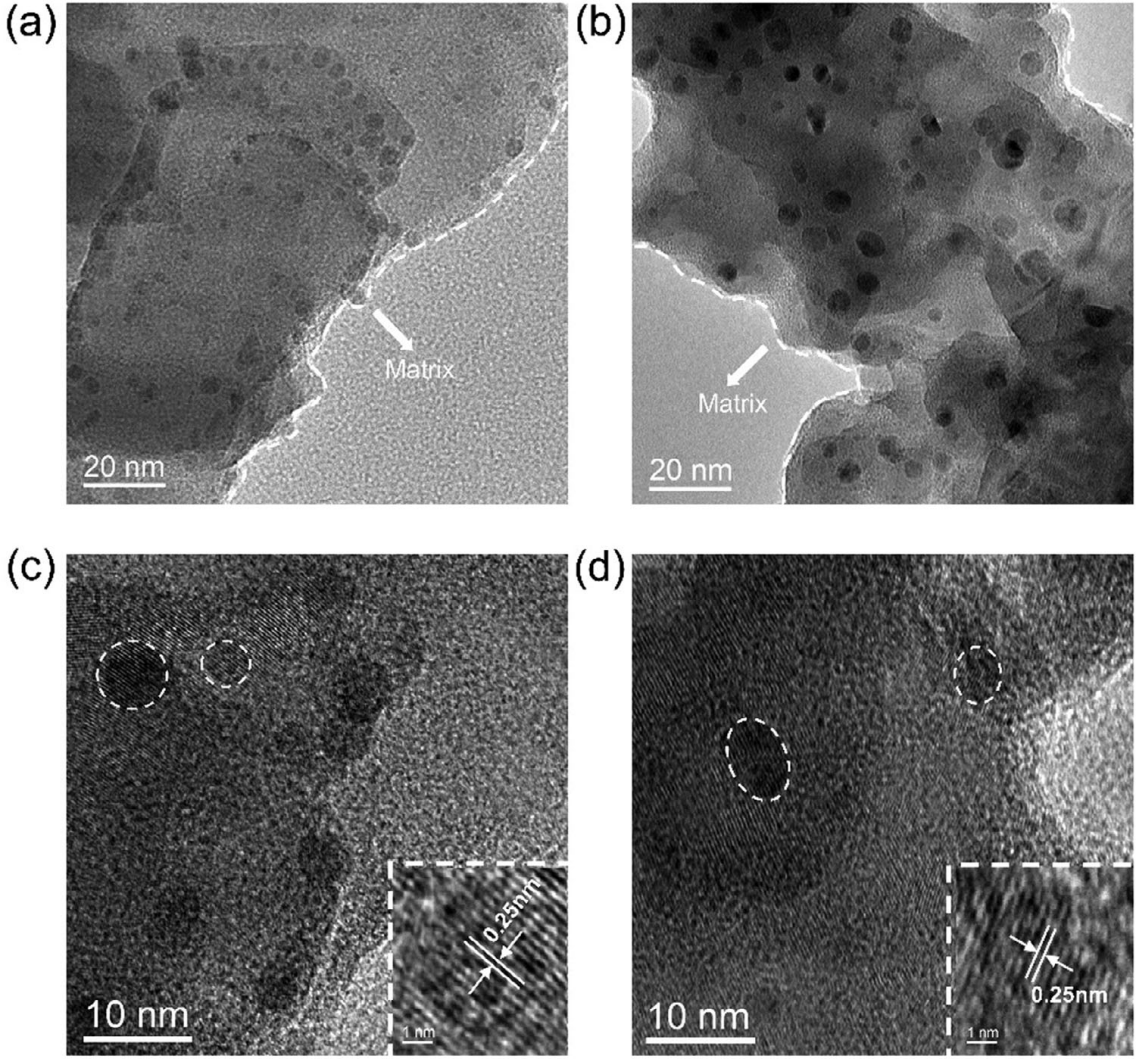
Transmission electron microscopy (TEM) images of (a, c) m‐CDs/U@200, (b, d) o‐CDs/U@200 (insets: high‐resolution TEM images).

To investigate the distinct afterglow behaviors of the three composites, we conducted TEM measurements using m‐CDs/U@X and o‐CDs/U@X under reaction temperature at 200 °C as well. In **Figure** **5**a,b, their TEM images show uniformly distributed spherical particles with average particle sizes of 4.25 and 5.07 nm, respectively (Figure S25, Supporting Information). HR‐TEM images display that they have a similar lattice spacing to p‐CDs/U@X (Figure 5c,d). Furthermore, inspired by the aforementioned discussion, the XPS spectra of three components can be measured. As shown in Figure S26 (Supporting Information), all of their XPS N 1s spectra include three peaks at 398.5 eV for pyridinic N, 399.7 eV for pyrrolic N, and 400.8 eV for graphitic N.^[^ ^22^, ^34^ ^]^ By careful comparison, we found that p‐CDs/U@200 and o‐CDs/U@200 show higher contents of pyridinic N and pyrrolic N compared with those of m‐CDs/U@200 (Table S4, Supporting Information), resulting in the formation of a higher conjugation structure, which can be responsible for their distinct afterglow colors (Figures S27–S29, Supporting Information).^[^ ^42^, ^52^, ^53^ ^]^

To verify the critical role of urea pyrolysis products as a matrix in TDAC, we prepared p‐CDs, m‐CDs, and o‐CDs using the same experimental method with only phenylenediamine derivatives. These CDs showed no afterglow emission after the 420 nm light was turned off, as confirmed by their afterglow emission spectra (Figure S30, Supporting Information). Furthermore, a series of comparative experiments using p‐CDs/U@200 as an example were carried out with other matrices under the same experimental conditions. We found that after introducing p‐CDs into PVA and PVP to form thin films, no afterglow emission was observed when the 420 nm lamp was turned off (Figure S31, Supporting Information). It is noteworthy that the TDAC phenomenon was not observed in other matrices (boric acid, silica) under the same experimental conditions. These results demonstrate the significance of urea pyrolysis products in achieving time‐dependent afterglow under visible light excitation.

**Figure 6:**
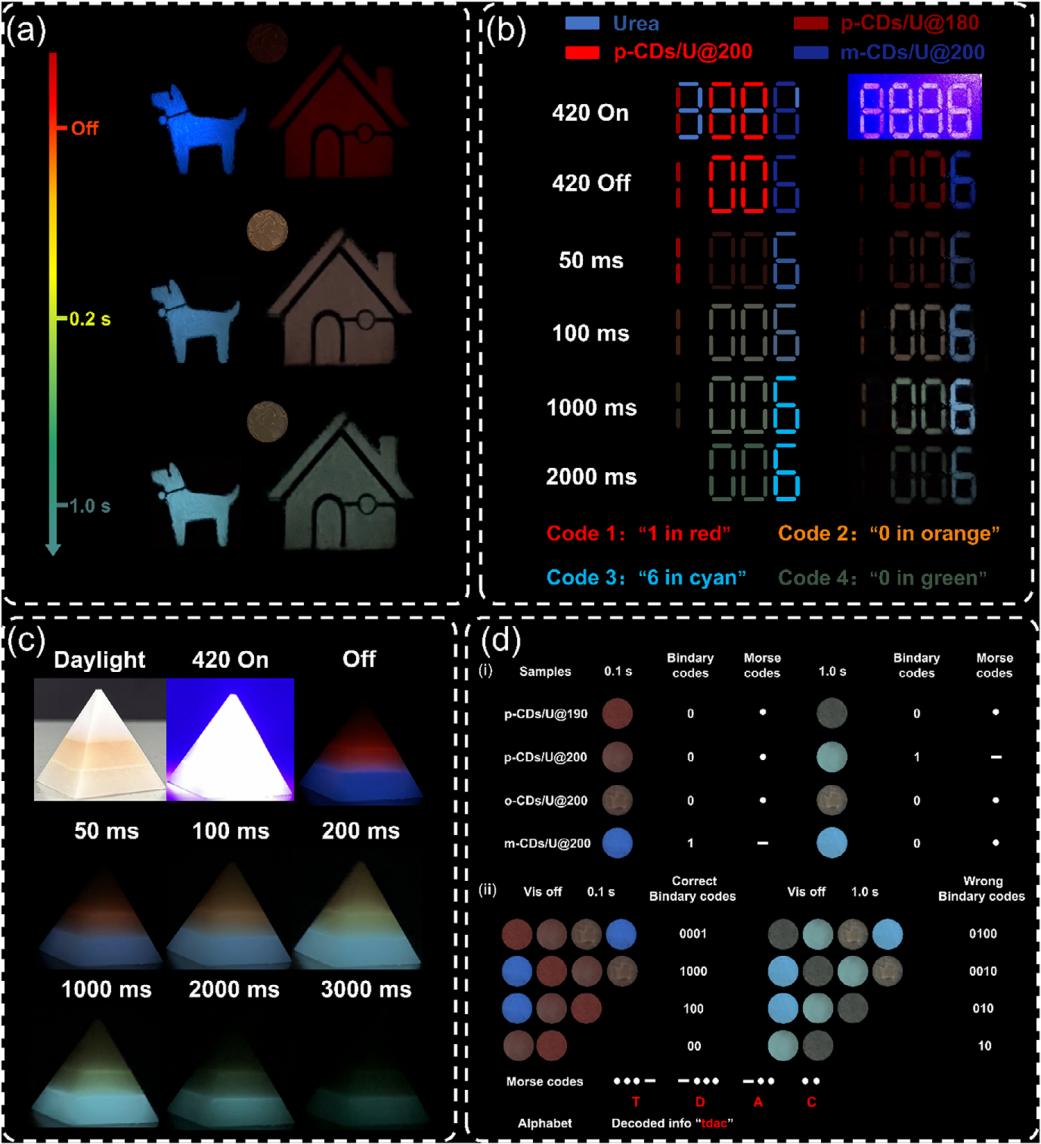
a) Anti‐counterfeiting pattern molds. b) Encryption and anticounterfeiting of numbers. c) Multi‐dimensional encryption of 3D artwork created using CDs/U composites. d) Morse code for messaging.

Owing to the multi‐color TDAC properties of CDs/U composites, they have great potential for anti‐counterfeiting and multi‐dimensional information encryption applications. First, we filled the three composites onto the specific pattern molds, where the house, dog, and sun areas were respectively modified with p‐CDs/U@200, m‐CDs/U@200, and o‐CDs/U@200 (**Figure** **6**a). After the Vis lamp was switched off, the house transitioned from red to green, the dog changed from blue to cyan, and the sun shifted from red to orange. The distinctive TDAC properties successfully achieve anti‐counterfeiting encryption in the time dimension, demonstrating their significant potential for smart anti‐counterfeiting applications. Second, we used pure urea as the interfering material, and the true information “1006” can be stored in the pattern “8888”. The digits “1”, “0”, and “6” were filled by p‐CDs/U@180, p‐CDs/U@200, and m‐CDs/U@200, respectively (Figure 6b). Under the Vis light, the false information “8888” was observed. When removing the Vis light, the digital message “1006” was decoded. As the fluorescence emission from urea disappears, its afterglow colors evolve with the codes “1 in red” at 100 ms, “0 in orange” at 100 ms, “6 in cyan” at 1000 ms, and “0 in green” at 2000 ms. This confirms that the correct information remains hidden until all four pattern‐, time‐, and color‐dependent codes are decoded. Furthermore, benefitting from the excellent water stability of CDs/U composites, three composites were successfully compatibilized with commercial epoxy resins. The mixed solutions were sequentially cast into the mold layer by layer to obtain a 3D artwork of a “pyramid” that can change color (Figure 6c). The three layers of the “pyramid” were composed of mixtures of o‐CDs/U@200, p‐CDs/U@200, and m‐CDs/U@200 composites with epoxy resin from top to bottom, respectively. After turning off the 420 nm lamp, the three levels of the pyramid show distinct color changes: the upper level from red to orange, the middle from red to green, and the lower from blue to cyan. The fabrication of 3D artwork with multicolor time‐dependent afterglow demonstrates the potential of CDs/U composites for smart optical encryption. Finally, the composites were also used in a Morse‐code‐like application to encrypt and decrypt information (Figure 6d). As illustrated in Figure 6d (i), we designated the afterglow colors blue and green as Morse code “1”, and all other colors as “0”. Based on this rule, the pattern shown in Figure 6d (ii) (left) was implemented to generate Morse code groups such as “0001”, “1000”, “100”, and “00”, corresponding to the Latin alphabet letters “T”, “D”, “A”, and “C”, respectively. These were successfully decrypted as “TDAC”. However, at 1.0 s, the message decrypts incorrectly due to signal degradation. The results demonstrate that CD‐based composites show promising potential for advanced dynamic anti‐counterfeiting and multi‐dimensional information encryption.

## 3 Conclusion

In summary, a novel and facile strategy is developed to achieve multi‐color tunable TDAC in the temporal dimension. Based on an in‐depth investigation of its time‐dependent mechanism, we obtain the following conclusions: the abundant surface states on CDs determine the short‐wavelength afterglows, while the high degree of conjugation is responsible for the long‐wavelength afterglows. The reaction temperature influences the matrix transformation from biuret to ammelide and then to CA. The significantly higher pyrrolic N content was observed when biuret and ammelide are used as matrices, which suggests that they can extend the conjugation degree of the nitrogen heterocycle structure. This gradually decreased conjugation degree is not conducive to long‐wavelength emission, leading to shorter lifetimes in the long‐wavelength region from biuret to ammelide and then to CA. Therefore, the time‐dependent afterglow can be effectively regulated by controlling the reaction temperature. Meanwhile, theoretical calculations reveal that the matrix plays a critical role in achieving efficient TADF emission: the narrower energy‐level difference (ΔE_S1T2_) between T_2_ and S_1_ and the increased SOC constant facilitate the RISC process. The excellent water stability of CDs/U composites originates from an extensive hydrogen‐bonding network. In this system, CDs not only interact strongly with the matrix through hydrogen bonds, but also establish a robust hydration hydrogen network through non‐freezing bound water. This dual protection mechanism more effectively prevents triplet energy quenching from water molecules and dissolved oxygen. Additionally, the distinct TDAC behaviors of the three composites were attributed to their different CDs formation mechanisms. Both p‐PD and o‐PD produce highly conjugated nitrogen‐heterocyclic structures during CDs formation, leading to long‐wavelength emission from red to green or orange. In contrast, when m‐PD is used as the precursor, the resulting CDs lack large π‐conjugated structures, resulting in short‐wavelength emission from blue to cyan. Finally, benefiting from the excellent TDAC characteristics of these composites, we have successfully developed multi‐mode anti‐counterfeiting and multi‐dimensional encryption.

## Conflict of Interest

The authors declare no conflict of interest.

## Supporting information



Supporting Information

Supplemental Movie 1

Supplemental Movie 2

Supplemental Movie 3

## Data Availability

The data that support the findings of this study are available from the corresponding author upon reasonable request.
