# Supplementary material for: Visible‐Light‐Activated Multi‐Color Tunable Time‐Dependent Afterglow Triggered by Variable Conjugation Effects via the Transformation of Matrix
Source: Adv Sci (Weinh). 2025 Aug 24;12(43):e10317. doi: 10.1002/advs.202510317 (PMC12631833; doi:10.1002/advs.202510317)
Supplement: Supplementary file 1 — Supporting Information [file ADVS-12-e10317-s002.docx]

Supporting Information

Visible-light-activated Multi-color Tunable Time-dependent Afterglow Triggered by Variable Conjugation Effects via the Transformation of Matrix

Hongpu Xin, Yunze Huang, Weibo Zhang, Peng Li,* and Huanrong Li*

H. Xin, Y. Huang, W. Zhang, P. Li, H. Li

Tianjin Key Laboratory of Chemical Process Safety, Hebei Key Laboratory of Functional Polymers, School of Chemical Engineering and Technology, Hebei University of Technology, Tianjin 300401, P. R. China

E-mail: lipeng@hebut.edu.cn; lihuanrong@hebut.edu.cn

**Supplementary Experimental Details**

**Materials**: All chemicals and materials were purchased from commercial sources and do not require further purification. p-Phenylenediamine (99%), m-Phenylenediamine (99%), o-Phenylenediamine (99%) were purchased from Macklin Biochemical Technology Co., Ltd (Shanghai, China). Urea (≥99%) was purchased from Kermel Chemical Reagent Co., Ltd (Tianjin, China). Deionized water was purified by laboratory deionized water equipment.

**Preparation of CDs/U**: 3 g urea and 20 mg p-phenylenediamine were completely dissolved in 20 mL of deionized water in a beaker. Covered the beaker with aluminum foil to prevent water from evaporating too quickly and to prevent other objects from falling into the beaker and contaminating the sample. Then the beaker was transferred to an oven at 180, 190, 200, 240, or 280 °C and reacted for 6 hours. After the above product was cooled to room temperature, it was ground into powder. To eliminate any small molecules, put the powder into a dialysis bag (MW:1000 Da) and exposed to the deionized water for 24 h. Finally, the suspension was freeze-dried to obtain the desired product. Two other CDs/U composites were prepared using m-phenylenediamine and o-phenylenediamine as precursors by the same method, respectively. The three composites were named p-CDs/U@X, m-CDs/U@X, o-CDs/U@X, respectively (X represents the temperature, X=180,190,200,240, or 280).

**Measurement**: Photoluminescence spectra, afterglow spectra, afterglow decay curves spectra, variable-temperature afterglow spectra, and time-resolved emission spectra (TRES) were measured with an FLS 1000 spectrometer from Edinburgh Instruments. And the afterglow emission spectra were achieved by time-gated technology with a delay time (t_d_) of 0.5 ms. Photoluminescence Quantum yields (PLQYs) and afterglow quantum yields (AQYs) were measured on an FLS1000 spectrometer equipped with an integrating sphere under ambient conditions. Photos and videos were taken with an iPhone 14 Pro. UV-vis absorption spectrum was performed using a Japen Shimadzu UV-3600 spectrometer. Transmission electron microscopy (TEM) and high-resolution TEM (HR-TEM) images were obtained using an FEI Talos F200S field emission electron microscope. X-ray diffraction (XRD) was measured by a Bruker D8 diffractometer scanned at 6 °/min from 5° to 90°. X-ray photoelectron spectroscopy (XPS) was performed using an America Thermo Scientific ESCALAB 250Xi. Raman spectra were measured using a Renishaw inVia Reflex micro-Raman spectrometer (785 nm excitation) and a Japan Horiba LabRAM HR Evolution spectrometer (514 nm excitation). Fourier transform-infrared spectroscopy (FT-IR) spectra in the range of 4000-400 cm^-1^ were collected using a BRUKER TENSOR 27 spectrometer. The thermogravimetric analysis (TGA) was performed using a HITACHI STA200 simultaneous thermal analyzer under a N₂ atmosphere, with a heating rate of 10 °C/min from room temperature to 800 °C. Differential scanning calorimetry (DSC) was performed on a HITACHI DSC200 Instrument at a heating rate of 5 K min^-1^.

**Density Functional Theory Calculations**: All-electron density functional theory (DFT) calculations were performed using ORCA program suite 6.0.0.^[1]^ For geometry optimization calculations, B3LYP functional and the def2-SVP basis set were used, and the optimal geometry was determined. The DFT-D3 dispersion correction with BJ-damping was applied to correct the weak interaction to improve the calculation accuracy.^[2,3]^ The excited states and Spin-Orbit Coupling (SOC) calculations were performed with B3LYP functional and the def2-TZVP basis set. SOC calculation was performed by spin-orbit mean-field (SOMF) method.^[4]^ Natural transition orbitals (NTOs) analysis was performed by Multiwfn 3.8 (dev).^[5,6]^

**
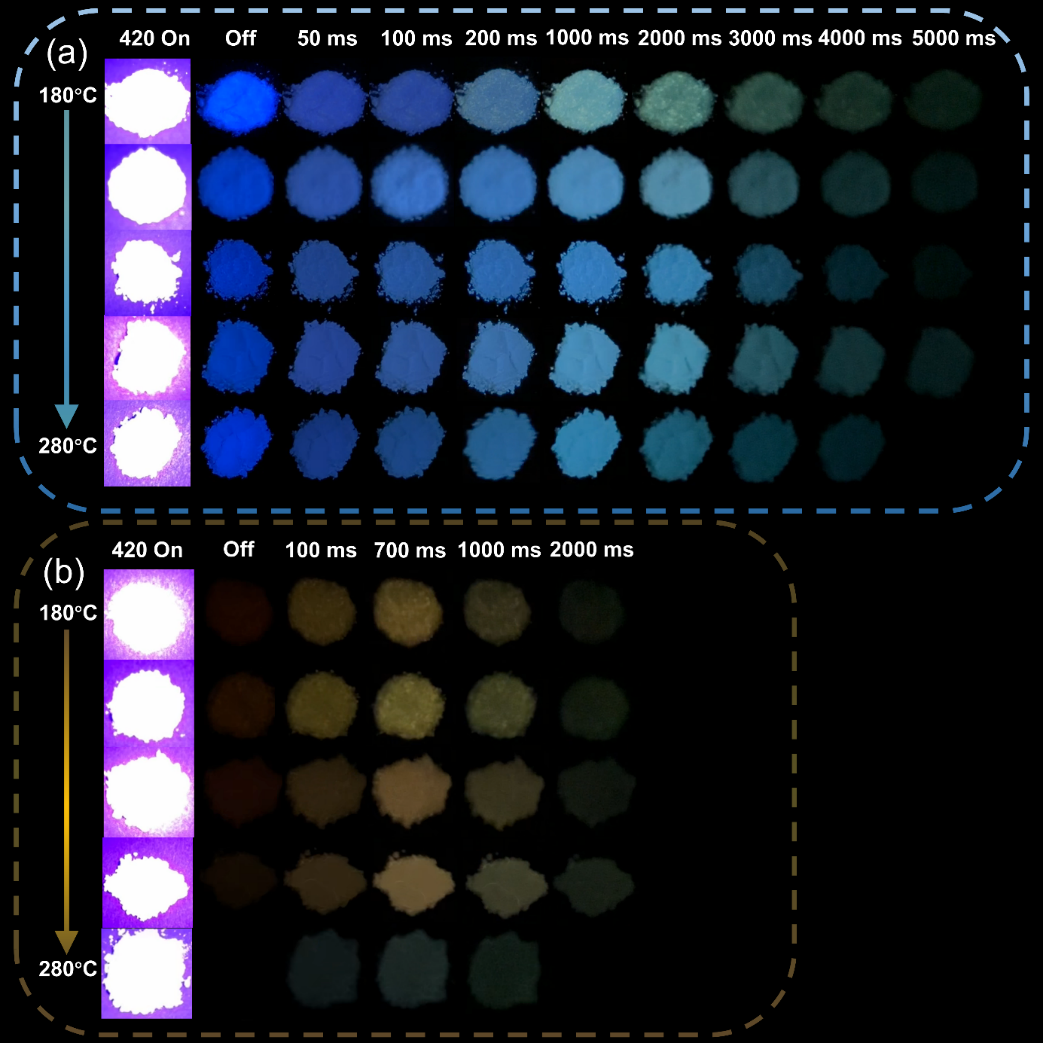
**

**Figure S1.** Digital photographs of CDs/U composites under 420 nm light irradiation and after being turned off, respectively.

**
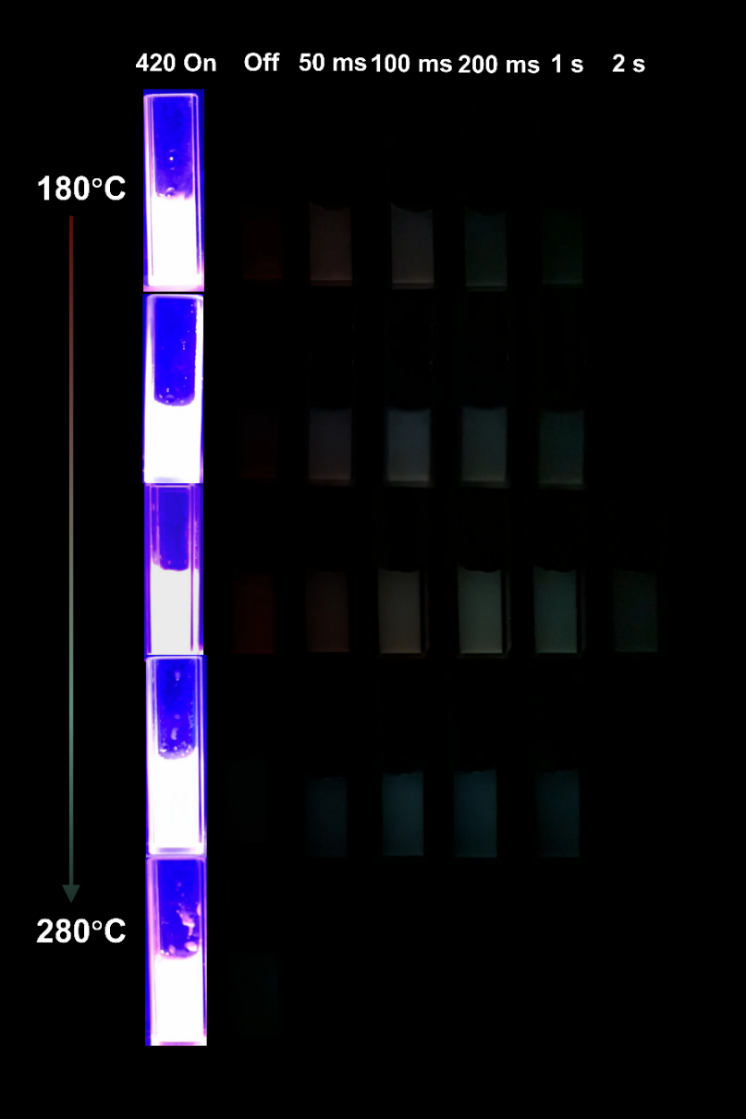
**

**Figure S2**. Photographs of aqueous solutions of p-CDs/U@X composites prepared at different temperatures under 420 nm excitation and after cessation of irradiation.


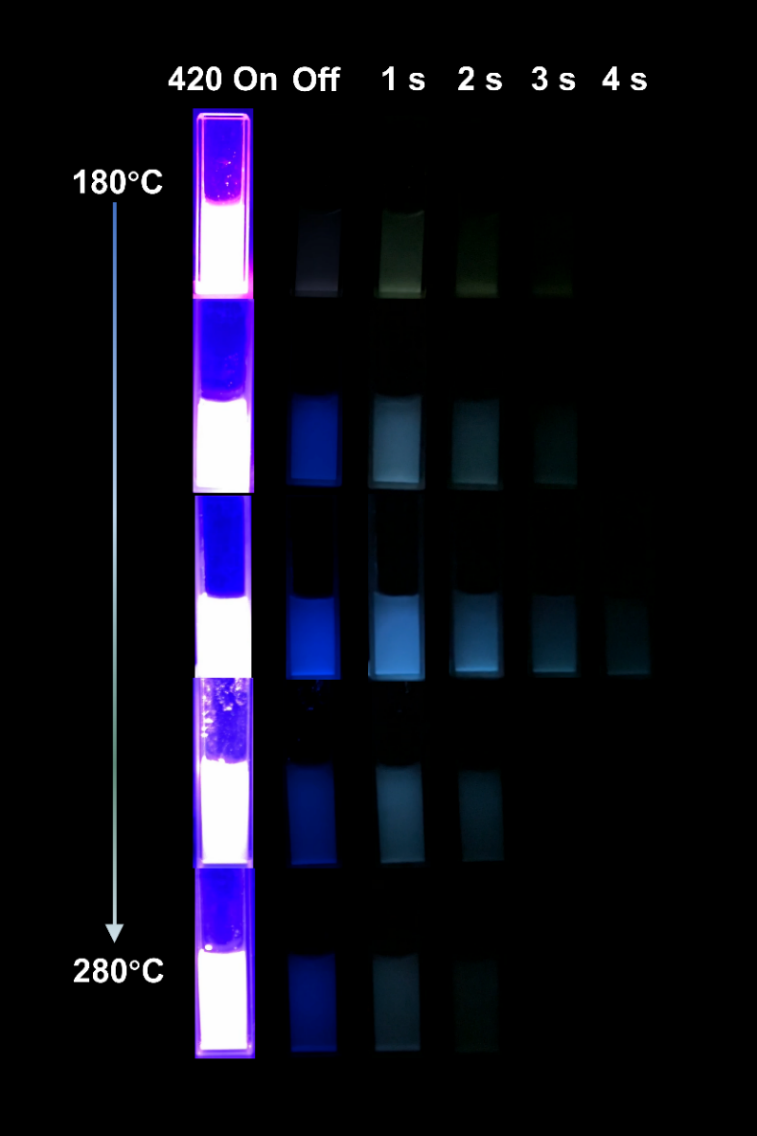


**Figure S3**. Photographs of aqueous solutions of m-CDs/U@X composites prepared at different temperatures under 420 nm excitation and after cessation of irradiation.


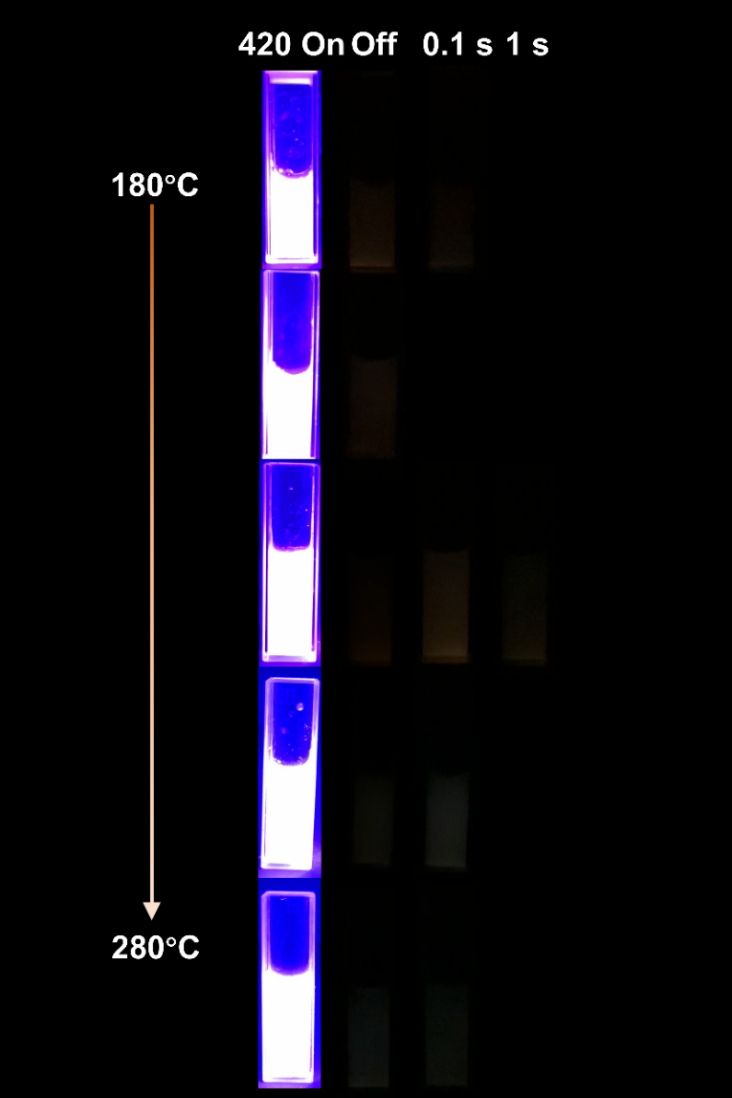


**Figure S4**. Photographs of aqueous solutions of o-CDs/U@X composites prepared at different temperatures under 420 nm excitation and after cessation of irradiation.

**
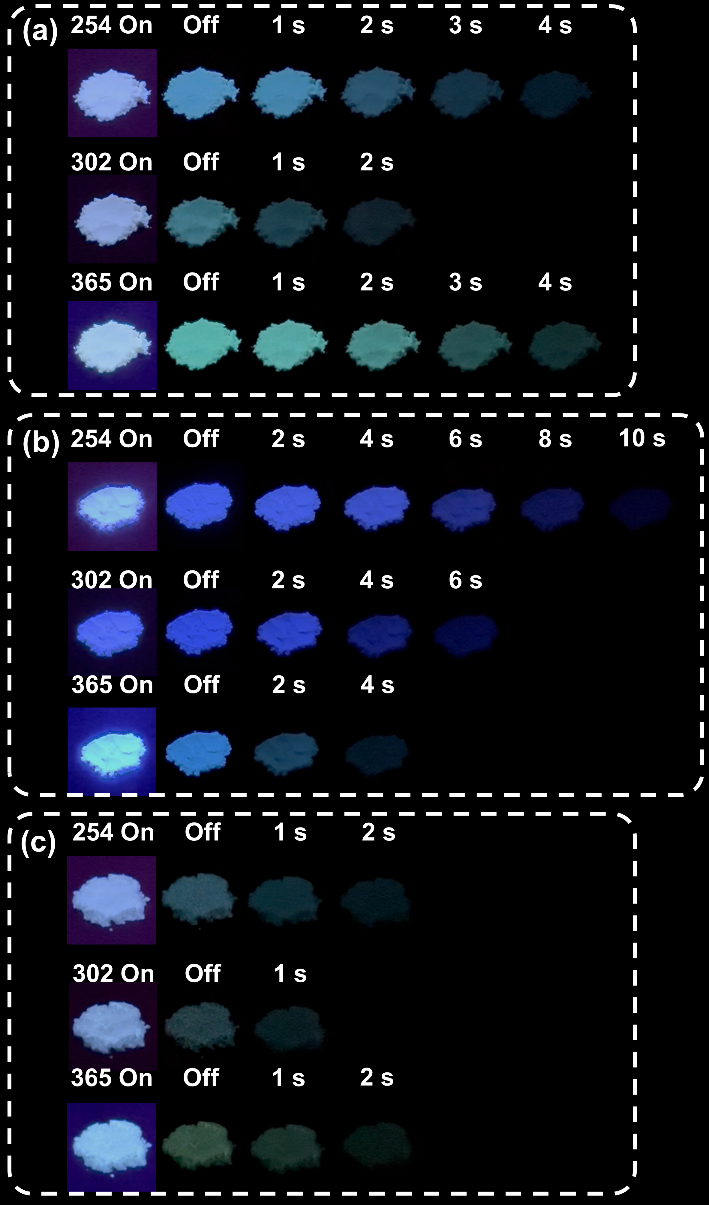
**

**Figure S5**. Digital photographs of (a) p-CDs/U@200, (b) m-CDs/U@200, and (c) o-CDs/U@200 before and after turning off under different excitation wavelengths.


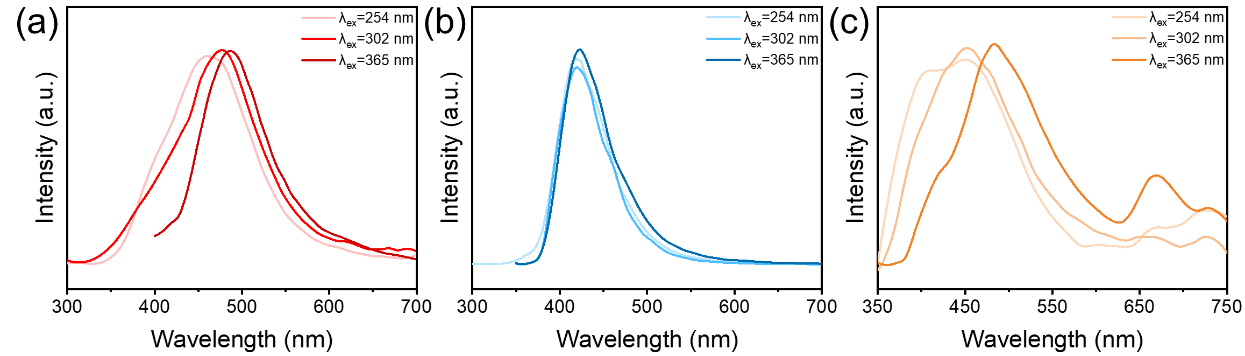


**Figure S6**. Digital photographs of a) p-CDs/U@200, b) m-CDs/U@200, c) o-CDs/U@200 before and after turning off under different UV excitation wavelengths.


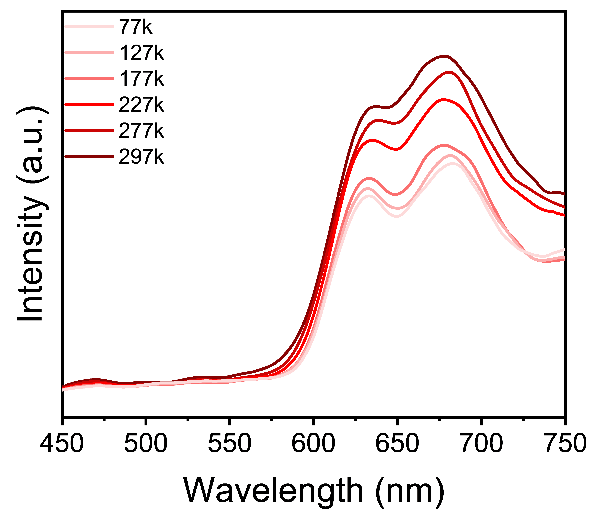


**Figure S7**. Variable-temperature afterglow spectra of p-CDs/U@200.

**Figure S8**. Variable-temperature afterglow spectra of m-CDs/U@200.


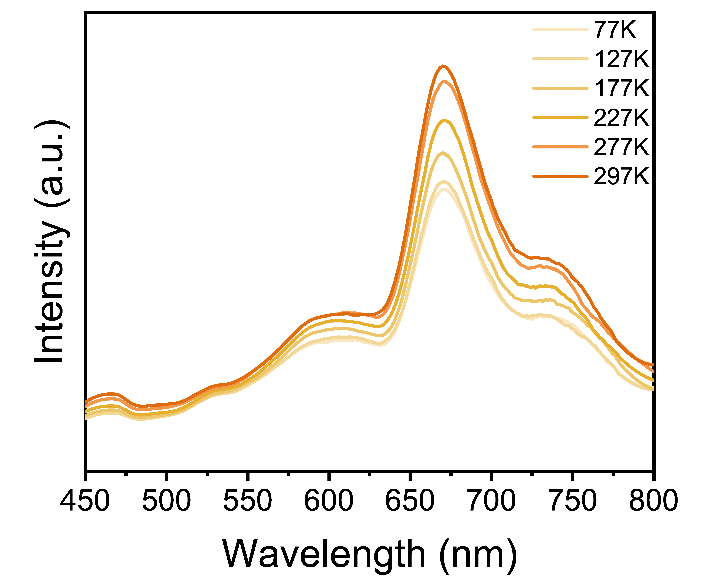


**Figure S9**. Variable-temperature afterglow spectra of o-CDs/U@200.

**Table S1**. Photoluminescence quantum yields (PLQYs) and afterglow quantum yields (AQYs) of the three composites at 420 nm excitation.

| **Sample** | **p-CDs/U@200** | **m-CDs/U@200** | | **o-CDs/U@200** |
| --- | --- | --- | --- | --- |
| PLQY | 18.02% | 22.25% | 39.40% | |
| AQY | 8.69% | 11.01% | 31.11% | |

**Table S2**. Comparison of our work with the reported CD afterglow materials.

|  | **Name** | ***τ_A_* (ms)** | ***Ф_A_* (%)** | **λ_ex_ (nm)** |
| --- | --- | --- | --- | --- |
| 1 | CNDs-RhB@silica^[7]^ | 910 | 3.56 | 400 |
| 2 | N-CDs/BA^[8]^ | 5 | 1.17 | 580 |
| 3 | R-CDs@BA^[9]^ | 16 | 1.6 | 400 |
| 4 | NIR-CDs@BA^[9]^ | 0.4 | 0.51 | 550 |
| 5 | Comp. 0-Comp. 4^[10]^ | 110-476 | 0.94-2.93 | 400 |
| 6 | CDs-I/B_2_O_3_^[11]^ | 423.5 | 17.61 | 440 |
| 7 | CDs@Mn-LEV^[12]^ | 1.81 | 5.7 | 420 |
| 8 | R-CDs_1mg_/BA^[13]^ | 583 | 0.6 | 450 |
| 9 | OR, Y, YG-CPDs^[14]^ | 426-511 | 1.5-6.3 | 401-466 |
| 10 | CDs@MnAPO-CJ50^[15]^ | 10.94 | 9.6 | 420 |
| 11 | CDs-N3^[16]^ | 278.03 | 16.78 | 420 |
| 12 | Br-CNQDs^[17]^ | 70 | 3.64 | 395 |
| 13 | Our Work | 48.68-304.31 | 8.69-31.11 | 420 |


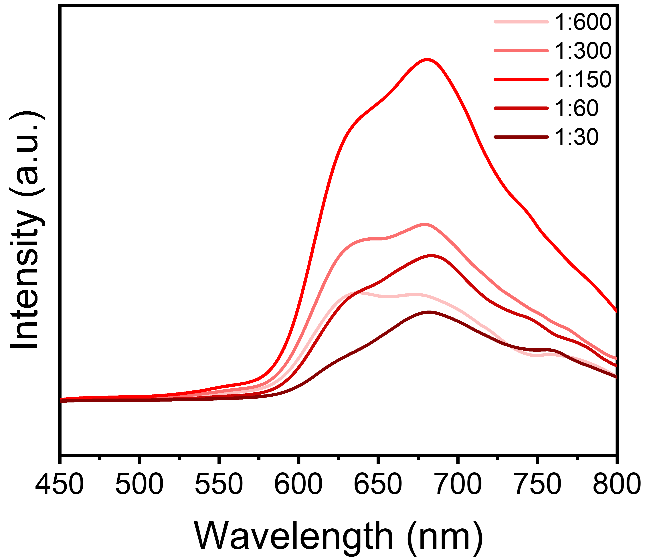


**Figure S10**. Afterglow emission spectra of samples prepared with p-PD and urea at different mass ratios.


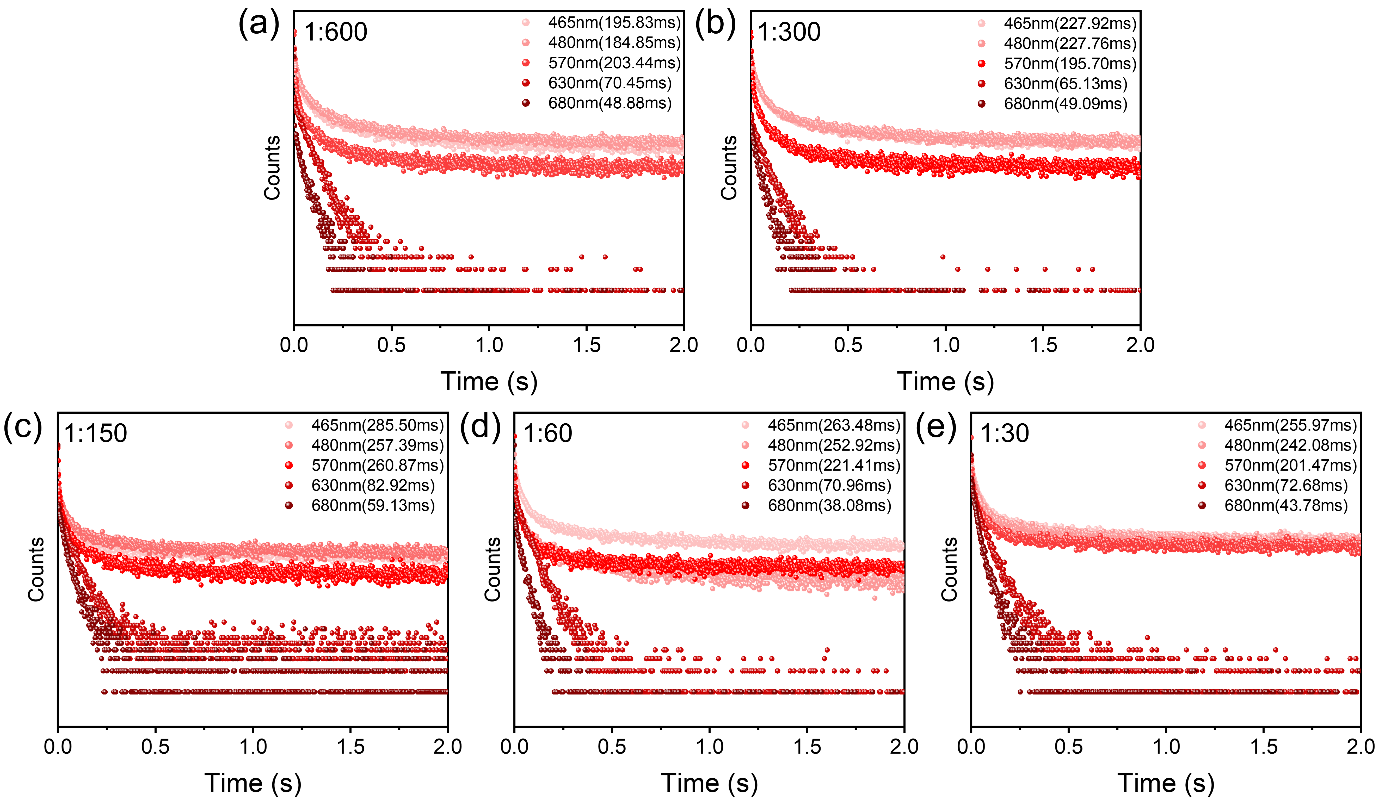
 **Figure S11**. Afterglow decay curves of samples prepared with p-PD and urea at different mass ratios.


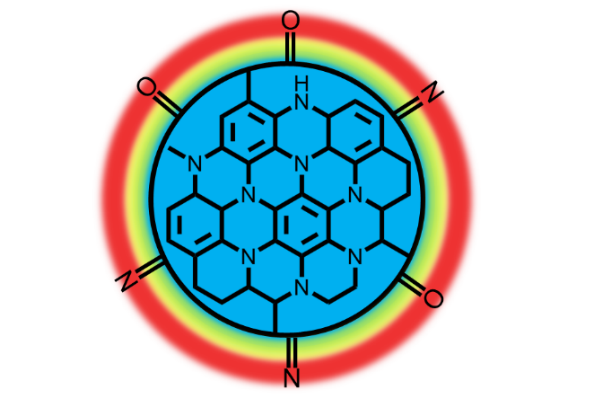


**Scheme S1**. Model for the dynamic multi-color TDAC of p-CDs/U@200.

**
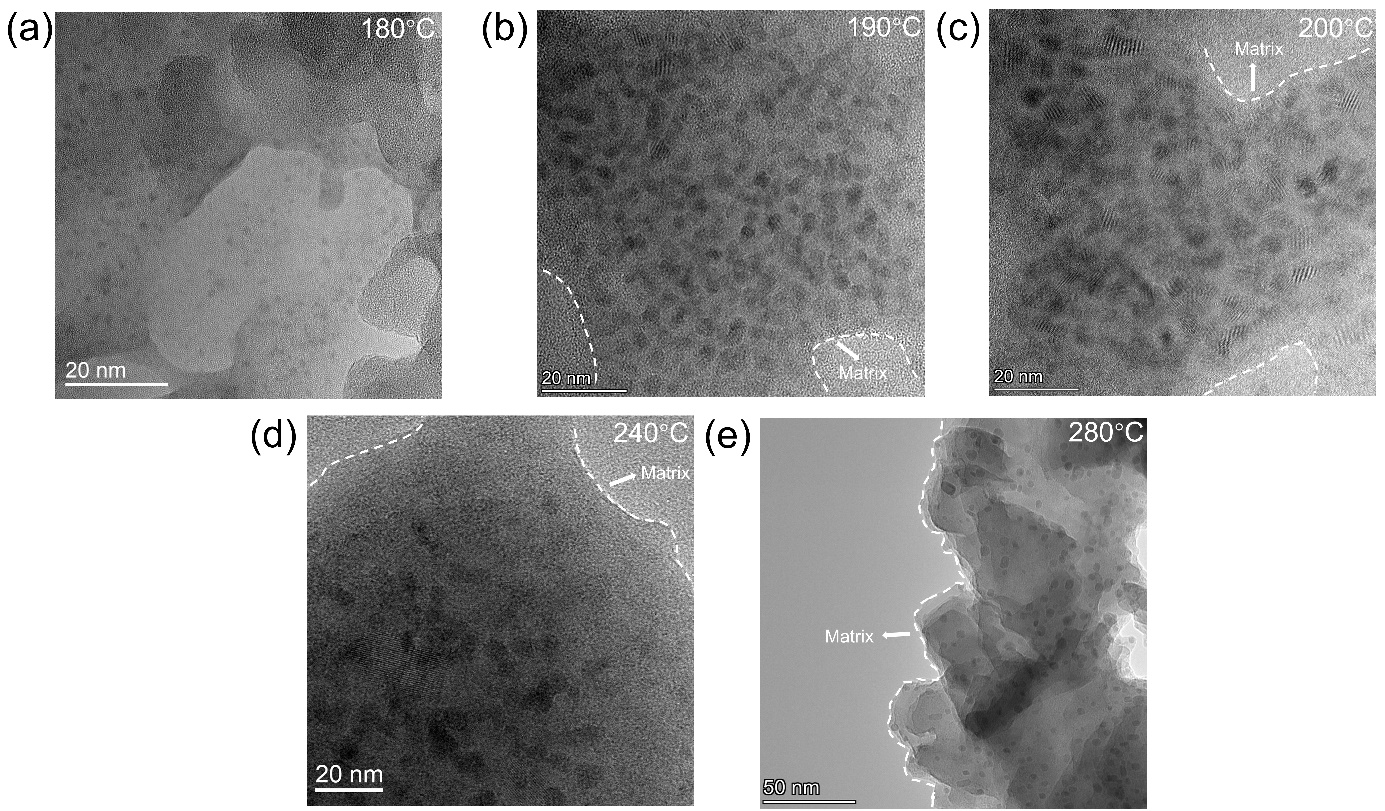
**

**Figure S12**. TEM images of (a) p-CDs/U@180, (b) p-CDs/U@190, (c) p-CDs/U@200, (d) p-CDs/U@240, and (e) p-CDs/U@280.


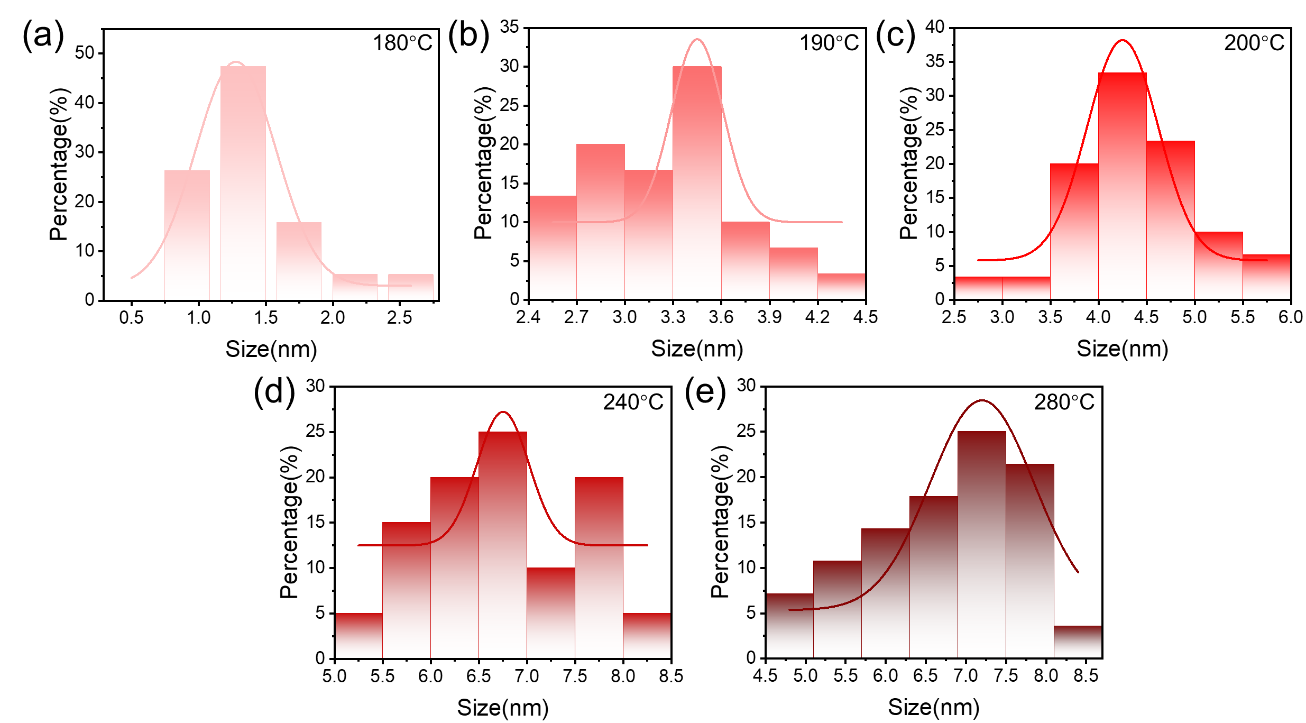


**Figure S13**. The size distribution obtained from TEM images of (a) p-CDs/U@180, (b) p-CDs/U@190, (c) p-CDs/U@200, (d) p-CDs/U@240, and (e) p-CDs/U@280.

**
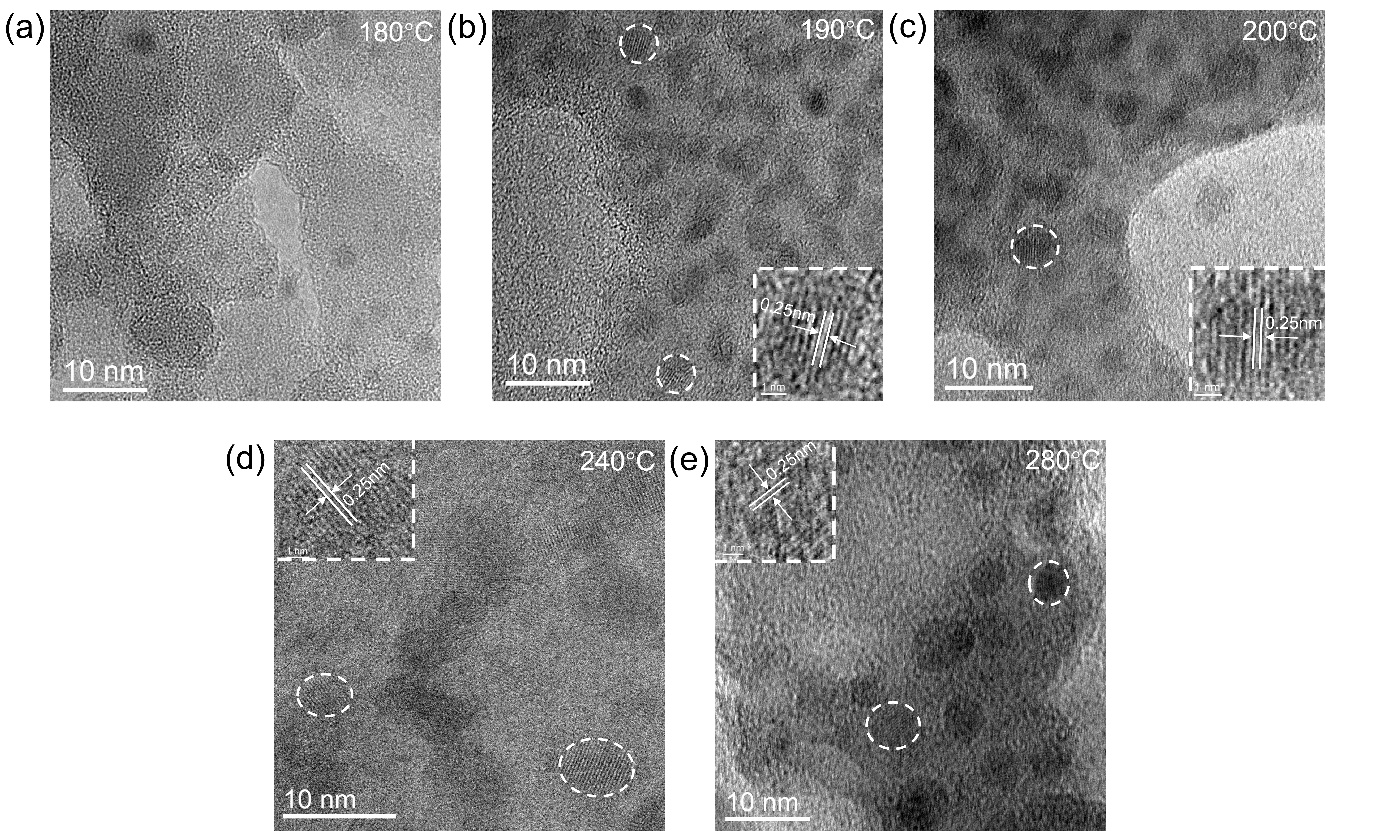
**

**Figure S14**. TEM images of (a) p-CDs/U@180, (b) p-CDs/U@190, (c) p-CDs/U@200, (d) p-CDs/U@240, and (e) p-CDs/U@280 (insets: high-resolution TEM images).


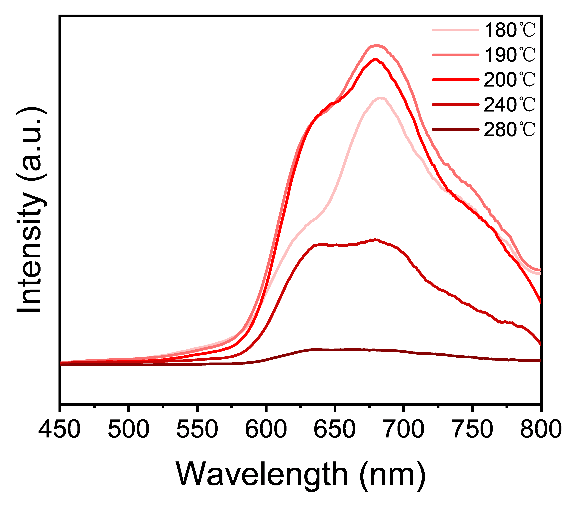


**Figure S15**. Afterglow spectra of p-CDs/U composites under 425 nm excitation.


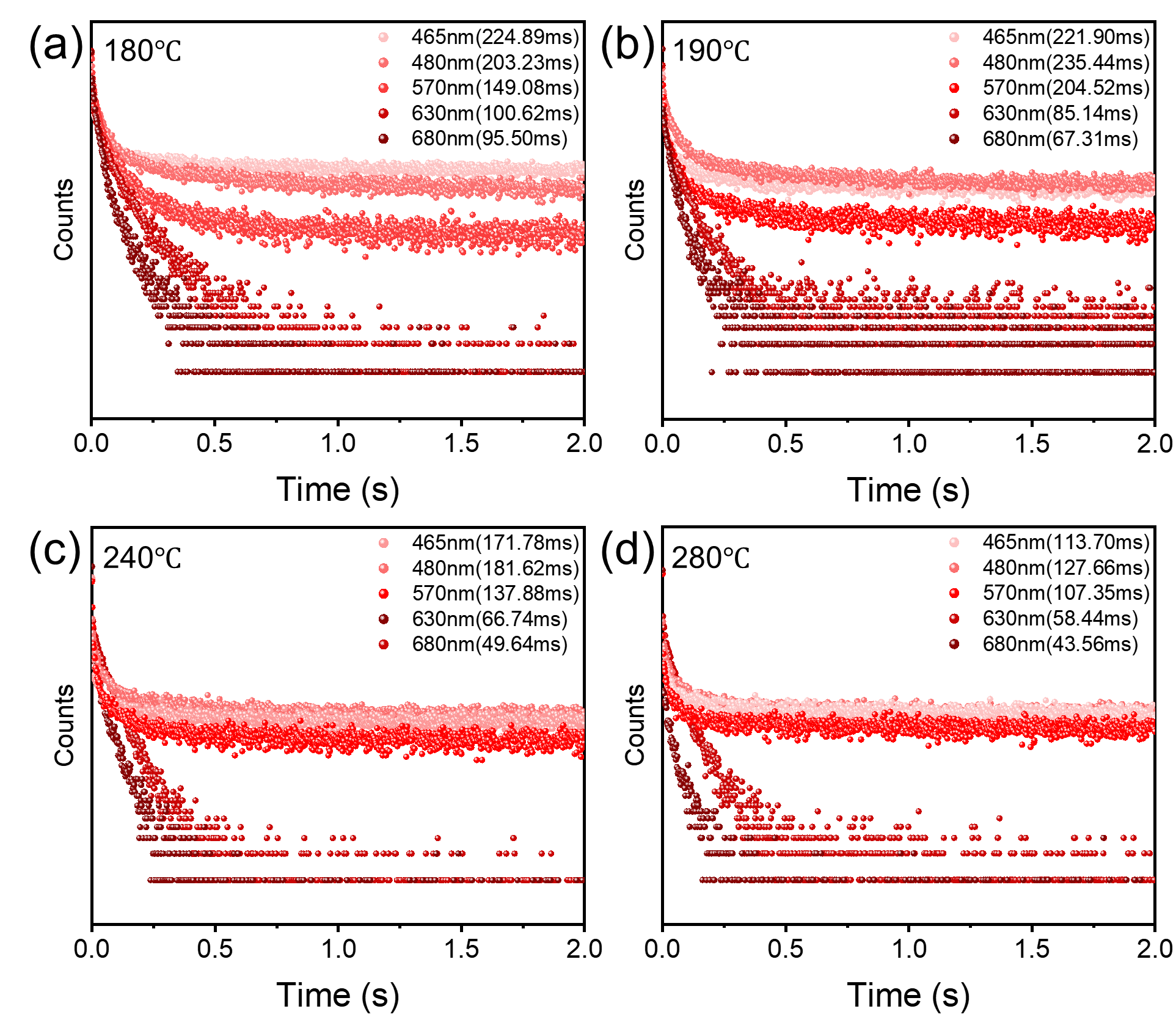


**Figure S16**. Afterglow decay curves spectra of (a) p-CDs/U@180, (b) p-CDs/U@190, (c) p-CDs/U@240 and (d) p-CDs/U@280 at 465 nm, 480 nm, 570 nm, 630 nm, and 680nm, respectively.


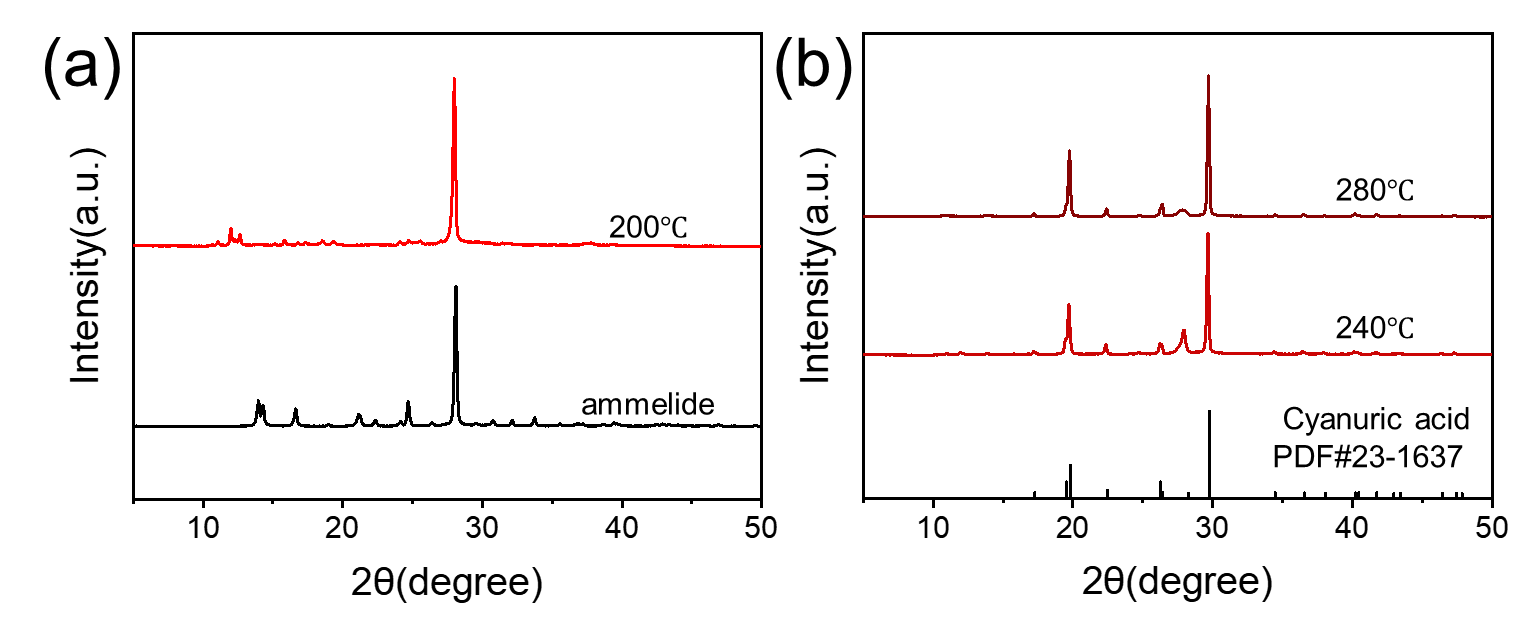


**Figure S17**. (a) XRD patterns of ammelide and p-CDs/U@200. (b) XRD patterns of Cyanuric acid (CA), p-CDs/U@240, and p-CDs/U@280.


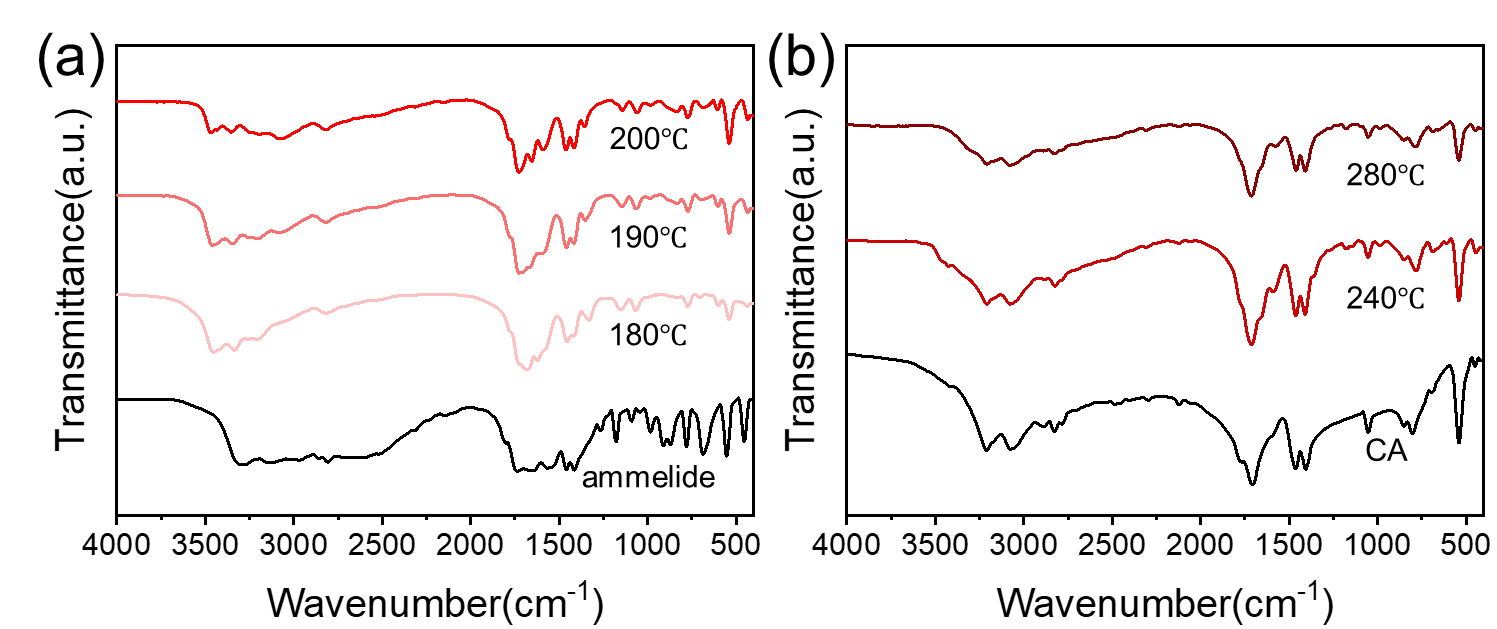


**Figure S18**. (a) FT-IR spectra of ammelide, p-CDs/U@180, p-CDs/U@190, and p-CDs/U@200. (b) FT-IR spectra of Cyanuric acid (CA), p-CDs/U@240, and p-CDs/U@280.


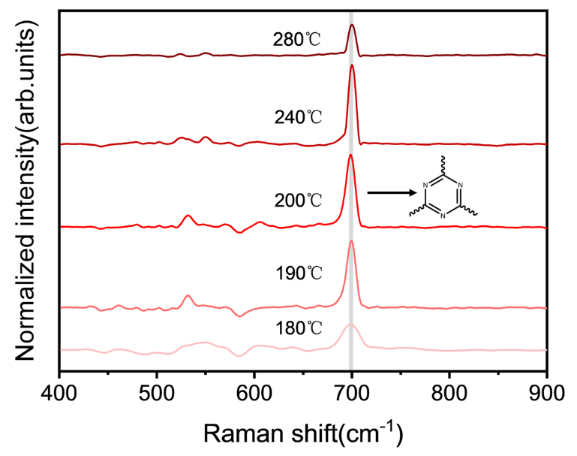


**Figure S19**. Raman spectra of p-CDs/U@X composites under 785 nm excitation.

**
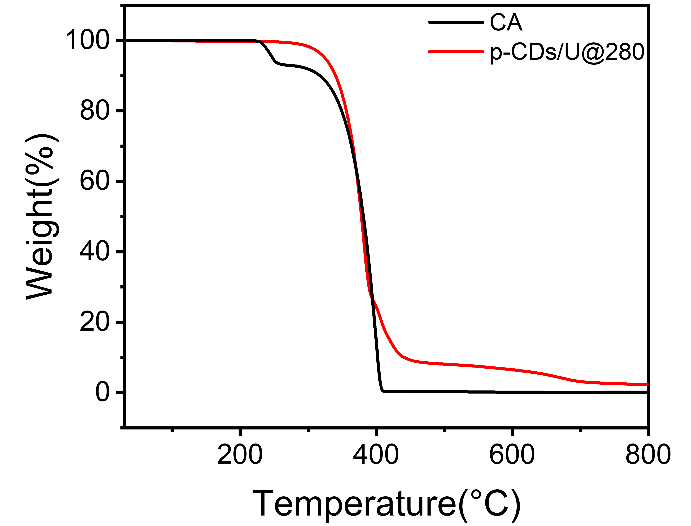
**

**Figure S20**. TGA curves of CA and p-CDs/U@280.

**
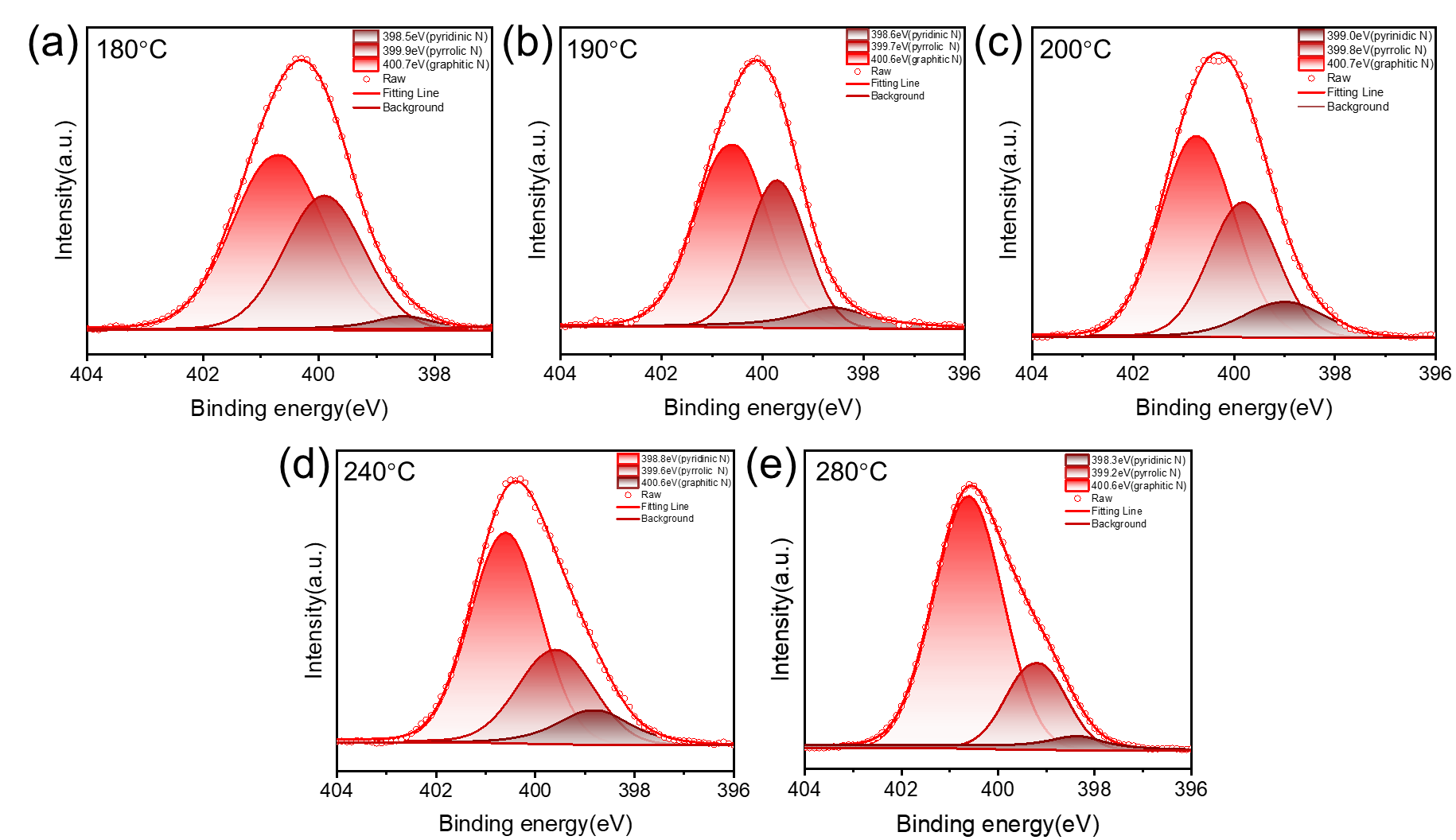
**

**Figure S21**. High-resolution N 1s X-ray photoelectron spectra of p-CDs/U@X composites.

**Table S3**. XPS data analyses of the N 1s spectra of p-CDs/U@X composites.

|  | pyridinic N (%) | pyrrolic N (%) | graphitic N (%) |
| --- | --- | --- | --- |
| 180°C | 4.70 | 38.22 | 57.09 |
| 190°C | 8.85 | 35.93 | 55.22 |
| 200°C | 12.08 | 34.50 | 53.43 |
| 240°C | 10.75 | 29.77 | 59.49 |
| 280°C | 9.06 | 21.01 | 69.63 |

**Discussion 1**

We propose a plausible mechanism in which two p-phenylenediamine (p-PD) molecules initially condense to form 2,7-phenazinediamine (2,7-DAP) during the hydrothermal synthesis of CDs using p-PD as the precursor. With prolonged the reaction time, the DAP structure undergoes crosslinking and polymerization in both horizontal and vertical dimensions, ultimately forming a high conjugation structure. While the presence of the matrix will further crosslink with p-CDs, expanding its conjugated nitrogen heterocyclic structure (Figure S22), which accounts for the red emission. Additionally, Sun et al. reported that benzidine carbon dots exhibit time-dependent ultra-long red TADF.^[18]^ Based on their theoretical calculations, we performed modeling of p-CDs and p-CDs/U@200 systems (Figure 4e).


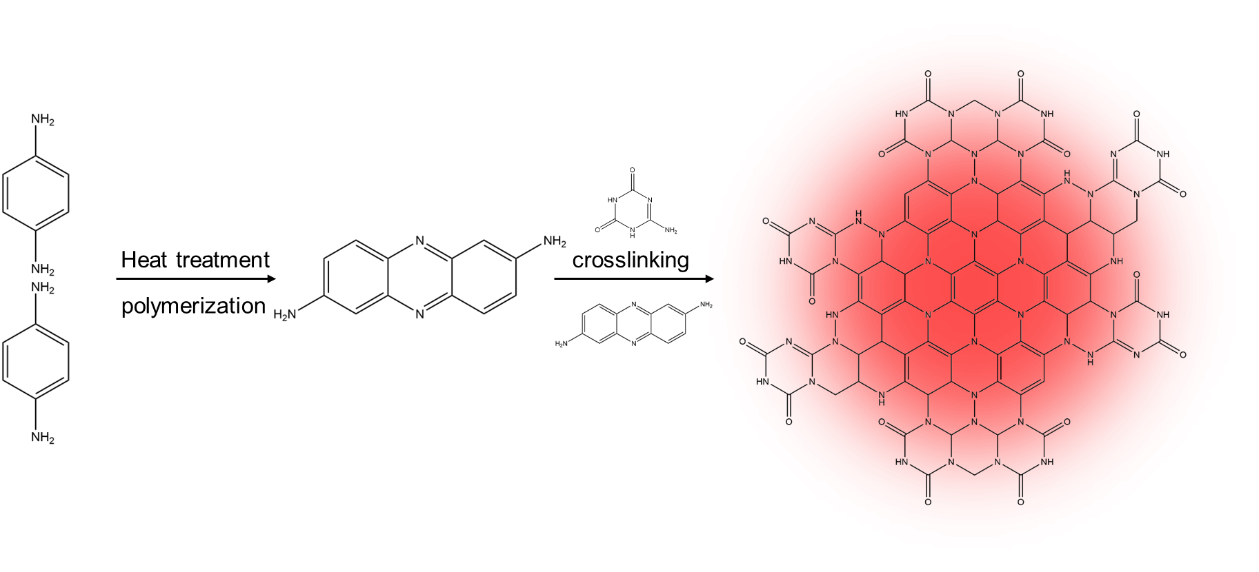


**Figure S22**. Possible process of formation of p-CDs/U@200.


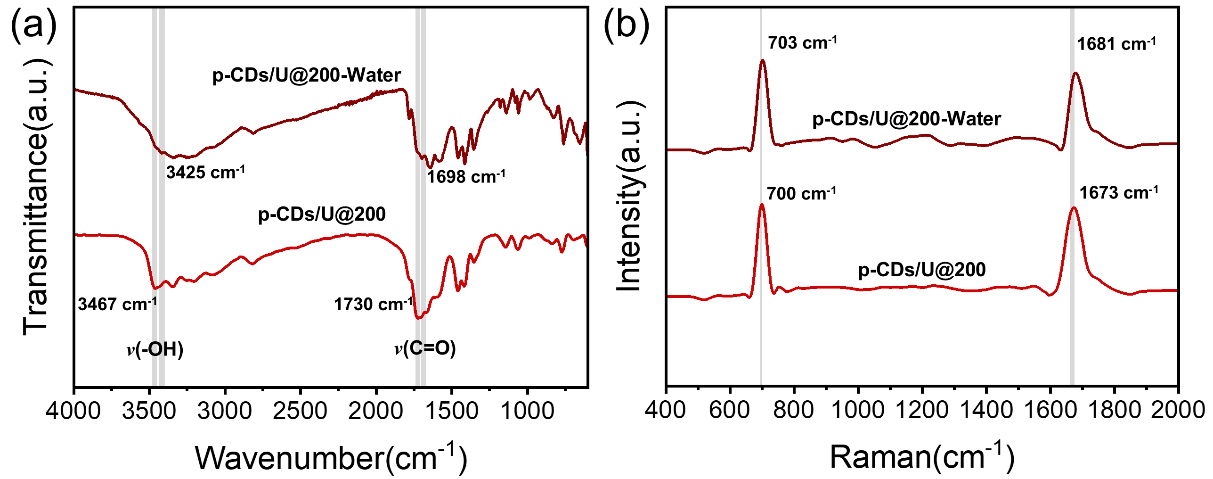


**Figure S23**. (a) FT-IR spectra of p-CDs/U@200 and p-CDs/U@200-Water. (b) Raman spectra of p-CDs/U@200 and p-CDs/U@200-Water.


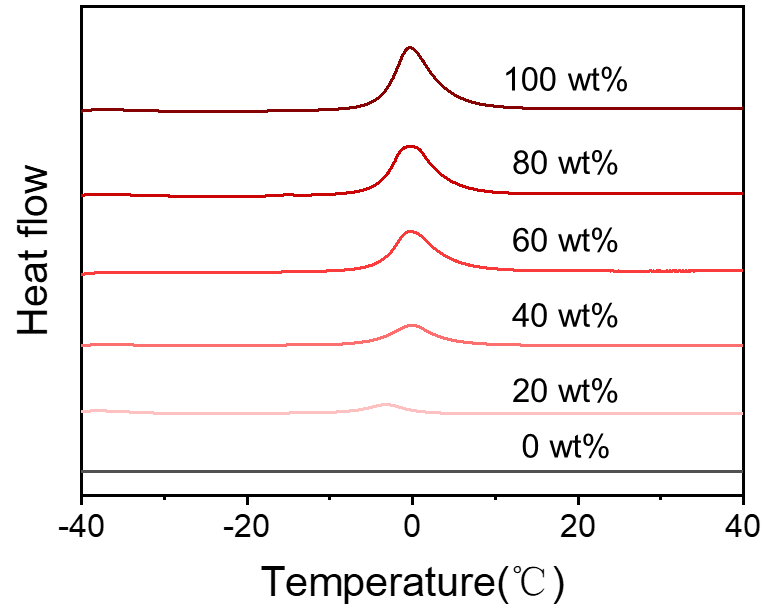


**Figure S24**. DSC-heating curves of p-CDs/U@200 systems with different water contents.


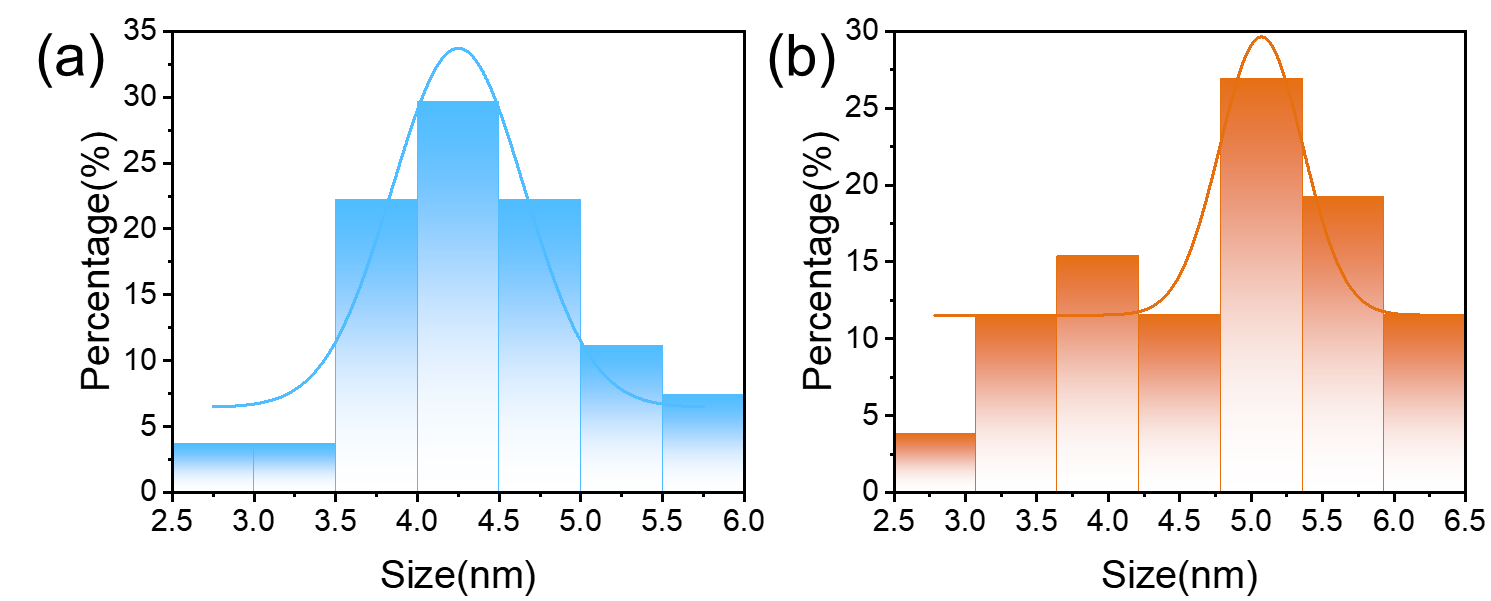


**Figure S25**. The size distribution obtained from TEM images of (a) m-CDs/U@200, and (b) o-CDs/U@200.


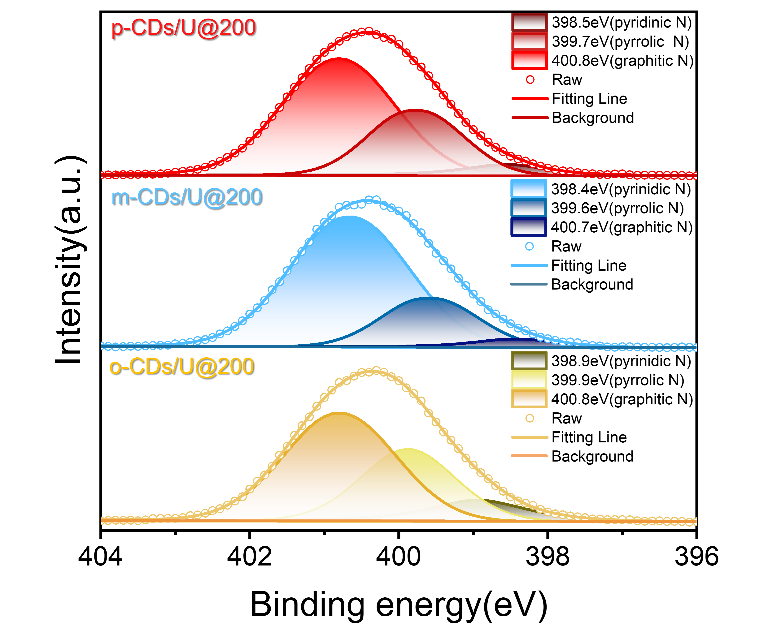


**Figure S26**. High-resolution N 1s X-ray photoelectron spectra of CDs/U@200 composites.

**Table S4**. XPS data analyses of the N 1s spectra of CDs/U@200 composites.

|  | pyridinic N (%) | pyrrolic N (%) | graphitic N (%) |
| --- | --- | --- | --- |
| p-CDs/U@200 | 12.08 | 34.50 | 53.43 |
| m-CDs/U@200 | 5.24 | 21.94 | 72.82 |
| o-CDs/U@200 | 12.50 | 32.22 | 55.28 |


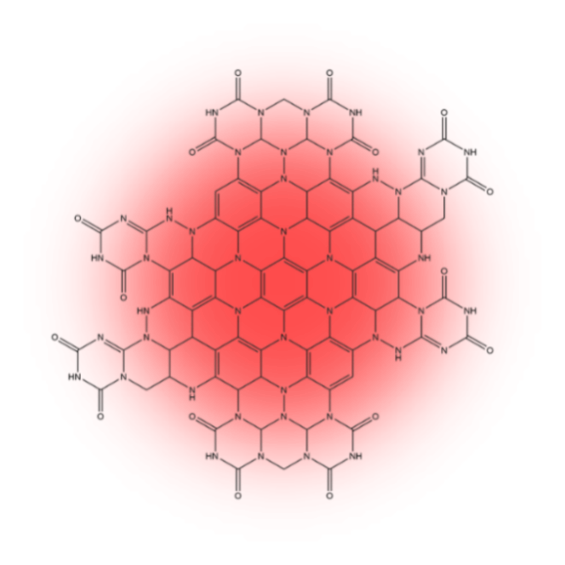


**Figure S27**. Possible structure of p-CDs/U@200.


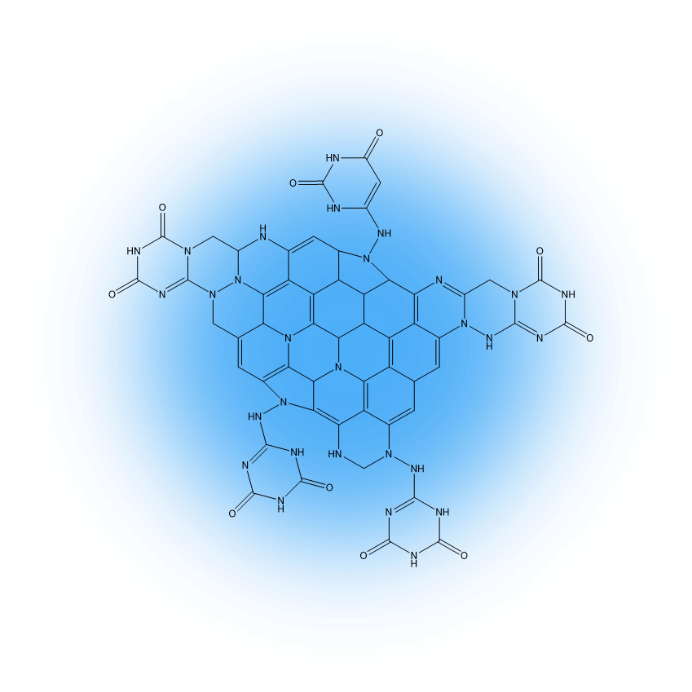


**Figure S28**. Possible structure of m-CDs/U@200.


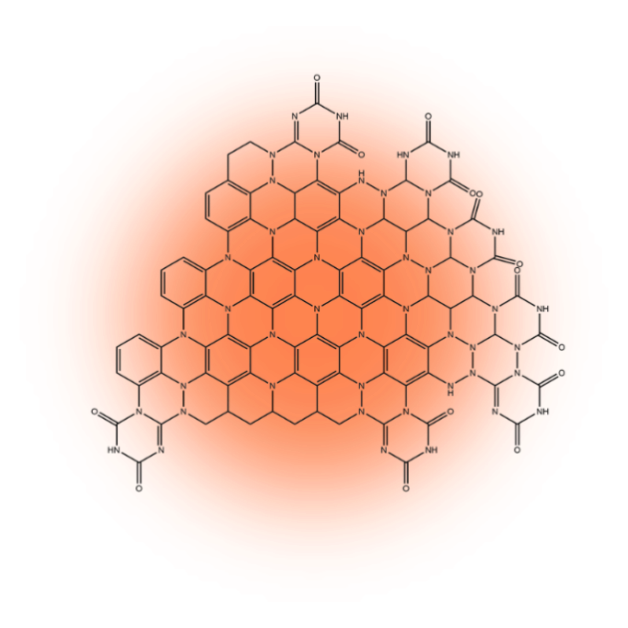


**Figure S29**. Possible structure of o-CDs/U@200.


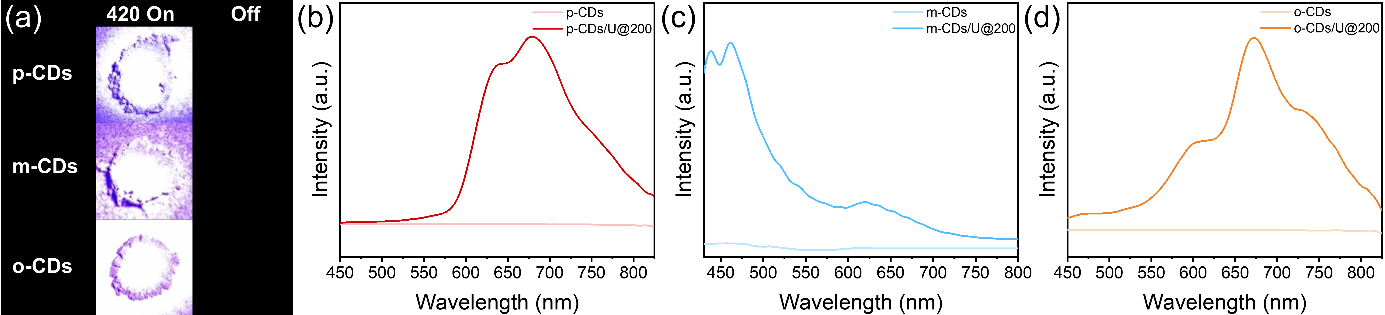


**Figure S30**. (a) Digital photographs of CDs under the same experimental conditions before and after turning off 420 nm light. The afterglow emission spectra of (b) p-CDs and p-CDs/U@200, (c) m-CDs and m-CDs/U@200, and (d) o-CDs and o-CDs/U@200.


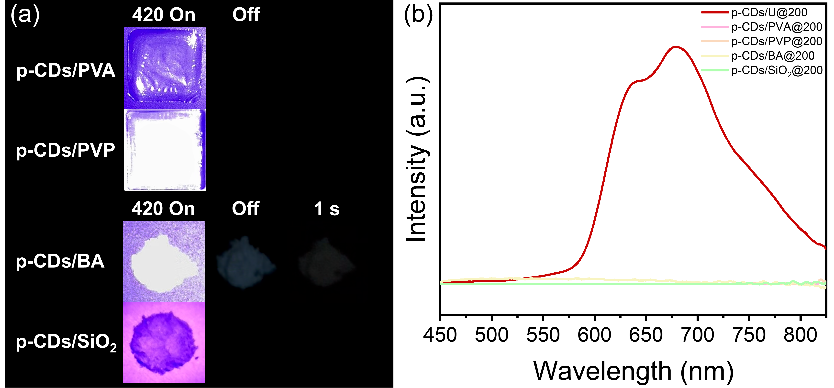


**Figure S31**. (a) Digital photographs of different matrices under the same experimental conditions before and after turning off 420 nm light. (b) The afterglow emission spectra of different matrices under the same experimental conditions under 420 nm excitation.

**References**

[1] F. Neese, *WIREs Comput. Mol. Sci.* **2018**, 8, e1327.

[2] S. Grimme, S. Ehrlich, L. Goerigk, *J. Comput. Chem.* **2011**, *32*, 1456-1465.

[3] S. Grimme, J. Antony, S. Ehrlich and H. Krieg, *J.Chem.Phys*. **2010**, 132, 154104.

[4] B. Hess, C. Marian, U. Wahlgren, O. Gropen, *Chem. Phys. Lett.* **1996**, 251, 365.

[5] T. Lu, F. Chen, *J. Comput. Chem.* **2012**, 33, 580-592.

[6] T. Lu, *J. Chem. Phys.* **2024**, 161, 082503.

[7] Y. Liang, Q. Cao, K. Liu**,** X. Peng, L. Sui, S. Wang, S. Song, X. Ying Wu, W. Zhao, Y. Deng, Q. Lou, L. Dong, C. Shan, *ACS Nano* **2021**, 15, 10, 16242-16254.

[8] Q. Zhao, C. Fan, H. Bu, J. Gao, L. Li, X. Yu, X. Yang, Z. Lu, S. Zhang, X. Zhang, *Chem. Eng. J.* **2025**, 511, 161986.

[9] Q. Li, D. Cheng, H. Gu, D. Yang, Y. Li, S. Meng, Y. Zhao, Z. Tang, Y. Zhang, J. Tan, S. Qu, *Chem. Eng. J.* **2023**, 462, 142339.

[10] Y. Liu, M. Al-salihi, Y. Guo, R. Ziniuk, S. Cai, L. Wang, Y. Li, Z. Yang, D. Peng, K. Xi, Z. An, X. Jia, L. Liu, W. Yan, J. Qu, *Light: Sci. Appl.* **2022**, 11, 163.

[11] W. He, X. Sun, X. Cao, *ACS Sustainable Chem. Eng.* **2021**, 9, 12, 4477-4486.

[12] B. Wang, Y. Mu, H. Zhang, H. Shi, G. Chen, Y. Yu, Z. Yang, J. Li, J. Yu, *ACS Cent. Sci.* **2019**, 5, 2, 349-356.

[13] M. Cheng, P. Wang, L. Cao, W. Dong, L. Li, R. Yan, *J. Lumin.* **2022**, 252, 119370.

[14] Z. Wang, J. Shen, B. Xu, Q. Jiang, S. Ming, L. Yan, Z. Gao, X. Wang, C. Zhu, X. Meng, *Adv. Opt. Mater.* **2021**, 9, 2100421.

[15] B. Wang, Y. Yu, H. Zhang, Y. Xuan, G. Chen, W. Ma, J. Li, J. Yu, *Angew. Chem., Int. Ed.* **2019**, 58, 18443-18448.

[16] W. Lin, Y. Wei, M. Feng, C. Li, B. Zhang, J. Kang, W. Liu, S. Guan, *ACS Appl. Opt. Mater.* **2025**, 3, 3, 712-719.

[17] Y. Kong, Y. He, Y. Wang, G. Song, *Nano Select* **2022**, 3, 402-410.

[18] J. Sun, Z. Sun, Z. Wang, N. Wang, Y. Han, L. Zhang, B. Zhang, X. Zhang, *Adv. Opt. Mater.* **2024**, 12, 2302542.
